# Automated Harmonic Signal Removal Technique Using Stochastic Subspace-Based Image Feature Extraction

**DOI:** 10.3390/jimaging6030010

**Published:** 2020-03-05

**Authors:** Muhammad Danial Bin Abu Hasan, Zair Asrar Bin Ahmad, Mohd Salman Leong, Lim Meng Hee

**Affiliations:** 1 Institute of Noise and Vibration, Universiti Teknologi Malaysia, 54100 Kuala Lumpur, Malaysia; salman.leong@gmail.com (M.S.L.); limmenghee@gmail.com (L.M.H.); 2 School of Mechanical Engineering, Universiti Teknologi Malaysia, 81310 Skudai, Johor, Malaysia; zair@utm.my

**Keywords:** harmonic removal, automated OMA (AOMA), operational modal analysis, stochastic subspace identification, stabilization diagram, clustering

## Abstract

This paper presents automated harmonic removal as a desirable solution to effectively identify and discard the harmonic influence over the output signal by neglecting any user-defined parameter at start-up and automatically reconstruct back to become a useful output signal prior to system identification. Stochastic subspace-based algorithms (SSI) methods are the most practical tool due to the consistency in modal parameters estimation. However, it will be problematic when applied to structures with rotating machines and the presence of harmonic excitations. Difficulties arise when automating this procedure without any human interaction and the problem is still unresolved because stochastic subspace-based algorithms (SSI) methods still require parameters (the maximum within-cluster distance) that are compulsory to be defined at start-up for each analysis of the dataset. Thus, the use of image-based feature extraction for clustering and classification of harmonic components and structural poles directly from a stabilization diagram and for modal system identification is the focus of the present paper. As a fundamental necessary condition, the algorithm has been assessed first from computed numerical responses and then applied to the experimental dataset with the presence of harmonic excitation. Results of the proposed approach for estimating modal parameters demonstrated very high accuracy and exhibited consistent results before and after removing harmonic components from the response signal.

## 1. Introduction

The present structural modal identification method, operational modal analysis (OMA), is widely and commonly used within various engineering fields, such as mechanical, aerospace, electrical and civil, due to its capability to implement economical and fast tests that rely solely on structural response signals induced by undetermined ambient excitations (operating loads, wind, turbulence, traffic) without affecting its operating conditions [1,2]. This means that OMA techniques have major advantages compared to classical experimental modal analysis (EMA), which requires input excitations for structural modal identification [3,4].

In recent years, the development of automated procedures for identifying modal parameters in operating conditions has become increasingly popular and stochastic subspace-based algorithms (SSI) methods have been selected as the most practical tool for this procedure due to the consistency in modal parameters’ estimation, especially under non-stationary noise excitations [5,6,7,8,9,10,11,12,13,14,15]. However, the use of subspace-based algorithms for OMA and structural health monitoring (SHM) will be problematic when applied to structures with rotating machines, due to the harmonic excitations. Harmonic components are sometimes considered as virtual modes in the identification and are potentially mistaken for being structural modes [16], and thus might lead to potentially bias the estimation of the actual modes where the standard automated OMA approaches cannot be applied in a straight-forward way [17,18].

It should be noted that harmonic components cannot, in general, be removed by simple filtering, as this would in most practical cases significantly change the poles of the structural modes and thereby their natural frequency and modal damping. Here, several indicators for the separation of structural and harmonic modes in output-only modal identification are proposed in Table 1 below.

In the context of a system subjected to random excitation combined with the steady-state signal, the subspace-based methods identify the harmonics as very lightly damped modes that one can filter in the mode selection process [28]. Difficulties arise when automating this procedure in the parametric method without the need of any human interaction because in traditional modal identification using the parametric method, the model order is often oversized in order to capture all physical modes in the frequency range of interest. Model oversizing is needed as models are often biased and do not include any noise modeling. The separation between physical and spurious modes involves a lot of interaction by a skilled analyst. Thus, a significant tool, such as a stabilization diagram, is needed to distinguish between physical and spurious modes. The selection of physical modes can be a complex task because it involves the setting of inconsistency thresholds for each modal parameter by the user. The development of automated OMA procedures marked a fundamental step toward the elimination of user intervention since traditional modal analysis requires a large amount of human intervention, particularly by an expert user. Since a lot of human intervention is for monitoring purposes, clustering tools are proposed to automate modal identification by discriminating physical poles from others. The current clustering tools require at least one user-defined parameter, the maximum within-cluster distance between representations of the same physical mode from different system orders and the supplementary adaptive approaches have to be employed to optimize the selection of cluster validation criteria [8,11,21,29]. In addition, the values for thresholds and parameters are inconsistent due to natural variations in modal properties of structures that come from damage or environmental influences that bring more difficulties to existing approaches [30]. 

In that case, a desirable solution is required to effectively identify and discard the harmonic influence over the output signal by neglecting any calibration or user-defined parameter at start-up and then automatically reconstructing back to become a useful output signal prior to system identification. Thus, the development of such a novel approach for an automated harmonic removal method in the SSI framework using image-based feature extraction for clustering and classification of harmonic components from structural poles and also for modal system identification is the focus of the present paper. As a fundamental necessary condition, the algorithm has been assessed first from computed numerical responses based on random white noise, acting on three shear-type frame structures, corrupted with noise and with the addition of harmonic excitation. Then, the original implementation is also applied to the experimental dataset with the presence of harmonic excitation.

The rest of the paper is organized as follows. Section 2 covers the steps of the theoretical formulation of an automated harmonic signal removal, while Section 3.1 discusses the preliminary analysis using the proposed approach on the simulated signal analysis using white noise input on a three-story frame and with the addition of harmonic excitation and multiples of it. Then, the comprehensive results and discussion of the experimental study on the three-story steel are presented in Section 3.1. Section 4 conclude the paper.

## 2. Materials and Methods

**Figure 1:**
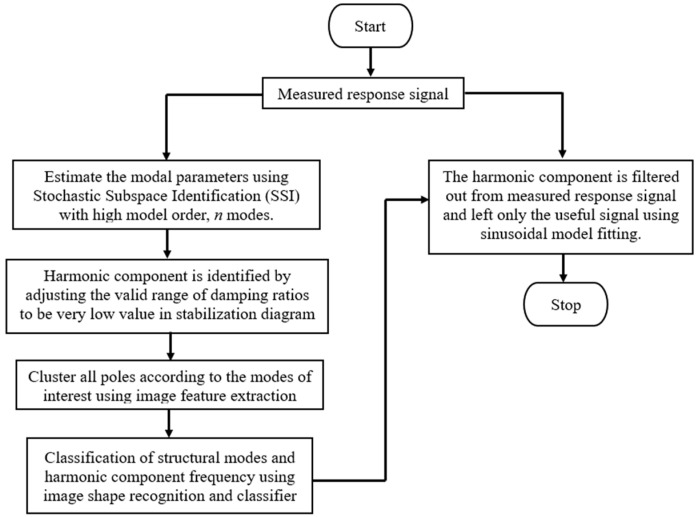
The steps of the automated harmonic signal removal technique.

The automated harmonic signal removal technique in OMA based on parametric methods consists of the following steps, as shown in Figure 1:

The steps required for the automated denoising technique are outlined in Figure 1. The first step is to estimate the modal parameters of the responses of the structure using the parametric method with a high model order. When the modal parameters are estimated, the stabilisation diagram can be constructed. Harmonic component can be identified by adjusting the valid limit range of modal damping ratios to be very low in the stabilization diagram. Then, image-based feature extraction is applied to cluster all poles according to modes of interest, all stabilized poles (structural or harmonic component). Classification of structural modes and harmonic component frequency is performed using image shape recognition and classifier. Finally, the identified harmonic frequency is filtered out from the measured response signal and only the useful signal using sinusoidal model fitting is left.

### 2.1. Stochastic Subspace Identification Technique (SSI)

Stochastic Subspace Identification (SSI) has been a recognized approach since the previous decade, primarily because of its user-friendly execution [31]. This paper is only concerned with correlation-driven SSI (COV-SSI), one of the SSI methods. The COV-SSI analyzes a stochastic state-space model from the response data of the structure [21] and a working algorithm almost similar to the Eigenvalue Realization Algorithm (ERA) [32]. Using SSI according to algorithm 1 for the principal component version requires a large model with 80 block rows of the half-block Hankel matrix for the estimation. The further details of its derivation are defined in the literature review. 

The initial step is to compute the output correlations as shown in Equation (1). $\left[R_{i}\right]$ indicates the correlation matrix at time lag *i* based on discrete data, as follows:

(1) $$\left[R_{i}\right]=\frac{1}{N-i}\left[Y_{\left(1:N-i\right)}\right]{\left[Y_{\left(i:N\right)}\right]}^{T}$$

where $\left[Y_{\left(1:N-i\right)}\right]$ is the data matrix *Y* with the last block rows, *i*, removed and $\left[Y_{\left(1:N-i\right)}\right]$ is the transpose data matrix with the first block rows, *i*, removed. Hence, each $\left[R_{i}\right]$ matrix gets dimensions l*l. The computed correlations at different time lags are then stored in the block Toeplitz matrix. The size of the Toeplitz matrix becomes n*n when estimating modal parameter with model order *n*. Thus, the subsequent Equation (2) should correct for the number of block rows *i*:

(2) $$li\geq \;n,\;i_{min}=x\frac{n_{max}}{l}$$

The magnitude, *x*, and maximum system order, *n*, were set as 2 and 50 modes, respectively. The next step is to calculate the singular value decomposition (SVD) of the block Toeplitz that can provide the unitary matrices [U] and [V]. The positive singular values are ranked in descendant order of the diagonal matrix [Σ] as in Equation (3) [33]:

(3) $$\left[T_{1|i}\right]=\left[U_{1}\right]\left[\Sigma_{1}\right]{\left[V_{1}\right]}^{T}\;=\left[O_{i}\right]\left[\Gamma_{i}\right]$$

To extract the dynamic response, the state matrix [A] needs to be obtained. This is done for each order from 1 to $n_{max}$. The observability matrix $\left[O_{i}\right]$ and the reversed controllability matrix $\left[\Gamma_{i}\right]$ are found by the factorization of $\left[T_{1|i}\right]$. The result of SVD of $\left[T_{1|i}\right]$ computed in Equation (3) can be used to find $\left[O_{i}\right]$ and $\left[\Gamma_{i}\right]$ by separating the SVD into two parts and using the identity matrix [I], as in Equations (4) and (5):

(4) $$\left[O_{i}\right]=\;\left[U_{1}\right]{\left[\Sigma_{1}\right]}^{1/2}{\left[I\right]}^{T}$$

(5) $$\left[\Gamma_{i}\right]=\;{\left[I\right]}^{-1}{\left[\Sigma_{1}\right]}^{1/2}{\left[V_{1}\right]}^{T}$$

Now that $\left[O_{i}\right]$ and $\left[\Gamma_{i}\right]$ have been obtained, the output influence matrix [C] and the state-output covariance matrix [G] can be computed. Matrix [C] is attained from the first row of $\left[O_{i}\right]$. Meanwhile, [G] is obtained from the last column of $\left[\Gamma_{i}\right]$. The normal Toeplitz matrix produces Equation (6):

(6) $$\left[T_{2|i+1}\right]=\left[O_{i}\right]\left[A\right]\left[\Gamma_{i}\right]$$

Resolving the eigenvalue problem for [A] produces the diagonal matrix [M] and the eigenvectors [Ψ], as in Equation (7):

(7) $$\left[A\right]=\left[\Psi \right]\left[M\right]{\left[\Psi \right]}^{-1}$$

The mode shapes of the system [Φ] are obtained from [Ψ] and [C], and the other modal parameters are attained from the eigenvalues $\mu_{m}$, which are found in the diagonal matrix [M]. The values are in discrete time and need to be transformed to continuous time, as in Equation (8):

(8) $$\lambda_{m}=\frac{\ln\left(\mu_{m}\right)}{\Delta t}$$

the complex $\lambda_{m}$ which contains the continuous time eigenvalues of each mode for the current order. This can be used to find the natural frequencies ($\omega_{n}$), damped modal frequencies ($\omega_{d}$) and modal damping ratio (ζ) for the r-th mode, as in Equations (9)–(11) as follows:

(9) $$\omega_{n,r}=\left|\lambda_{m,r}\right|$$

(10) $$\omega_{d,r}=Im\left(\lambda_{m,r}\right)$$

(11) $$\zeta_{r}=-\frac{Re\left(\lambda_{m,r}\right)}{\left|\lambda_{m,r}\right|}$$

The step of identifying the state matrix and the modal parameters are repeated for each order up until $n_{max}$ before being plotted in a stabilization diagram.

### 2.2. Harmonic Detection Using Stabilization Diagram

When the modal parameters are estimated using the SSI techniques, the stabilization diagram can be constructed in order to select the optimal State Space Dimension. This tool is a typical means to distinguish between stable, unstable and noise modes, which is performed by estimating poles with an increasing model order. The unstable and noise modes appear due to an oversized model system. The noise modes are caused by physical reasons, while the unstable modes are generated to ensure the mathematical description of the measured data. Theoretically, the stabilized physical modes can be identified by the vertical alignment of stable poles, while noise modes are scattered. This is based on the poles’ comparability with respect to the order of the given model with that obtained from a lower order model [21].

The natural frequencies and damping ratio of poles from two orders are compared using Equations (12) and (13) as follows [22]:

(12) $$\frac{\left|f\left(n-1\right)-f\left(n\right)\right|}{f\left(n-1\right)}<x$$

(13) $$\frac{\left|\zeta \left(n-1\right)-\zeta \left(n\right)\right|}{\zeta \left(n-1\right)}<y$$

For modes to be classified as stable, they must meet the specific requirements of the mode indicator with respect to the following thresholds which are set for variation between models of consecutive orders: natural frequency variation <1% [34], and modal damping ratio variation <5%. Meanwhile, a stabilization criterion for harmonic components is identified by adjusting the valid range of damping ratios to be of very low value, which is a modal damping ratio variation of less than 0.1%, as a limit for variations between models of consecutive orders, since damping ratio is an a priori indicator to distinguish between harmonics and structural poles. Generally, the damping ratios of real poles vary between 0.1% and 2%, while harmonic components exhibit the very low value of the damping ratios due to the appearance of the sharp peak. This information enables modes with negative and high damping to be eliminated [35]. These thresholds allow the clear distinction of vertical alignments as stable modes that represent the modes of vibration and both harmonic components and non-physical modes that can be filtered out. 

**Figure 2:**
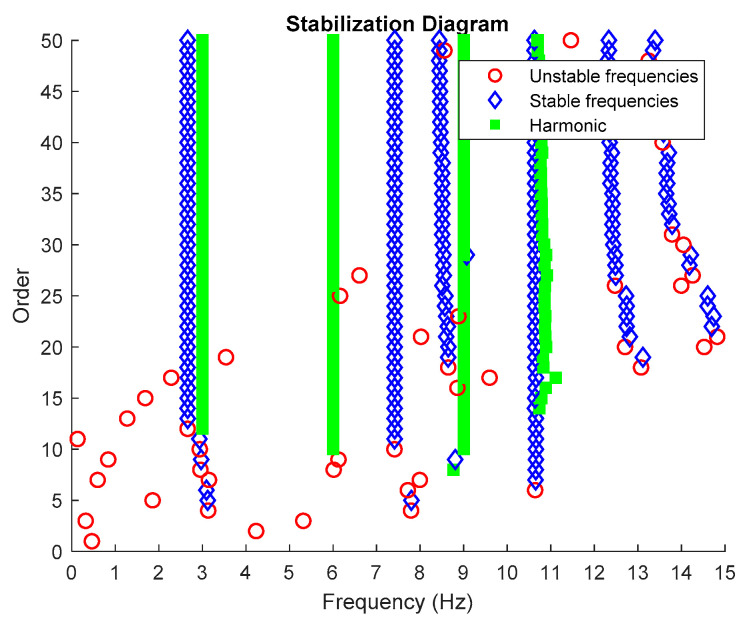
Stabilization diagram plot of three-story frame model from numerical simulations.

Then, all the distinguished poles displayed in the stabilization diagram plot are labelled into specific color and type of shape accordingly, as characterized in Table 2 below. Examples of an edited stabilization plot are shown in Figure 2 below. 

### 2.3. Clustering Using Image Feature Extraction

The procedure of using image clustering with respect to the similar physical pole of the stabilization diagrams is outlined below.

#### 2.3.1. Input Image

The process of image clustering requires the input image of the stabilization diagram that has been cut down into a certain interval frequency accordingly. In this case, the stabilization diagram was generated and displayed separately into every frequency according to a 0.01 interval as in Equations (14) and (15) below:

(maximum frequency)/0.01 = total images(14)

**Figure 3:**
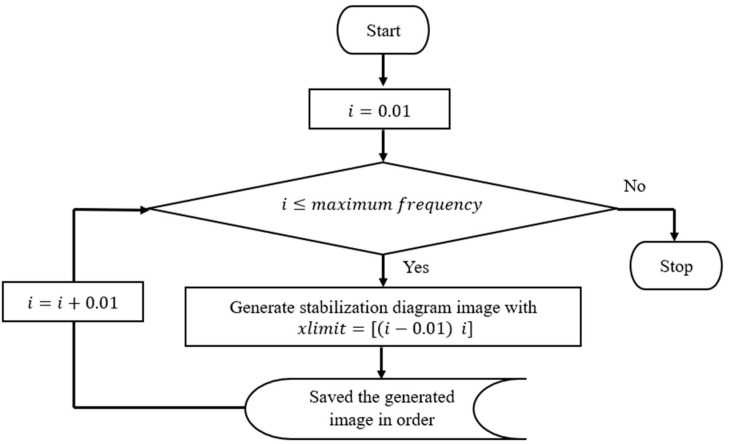
Flowchart process of generated input images from a stabilization diagram with a 0.01 Hz interval frequency.

**Figure 4:**
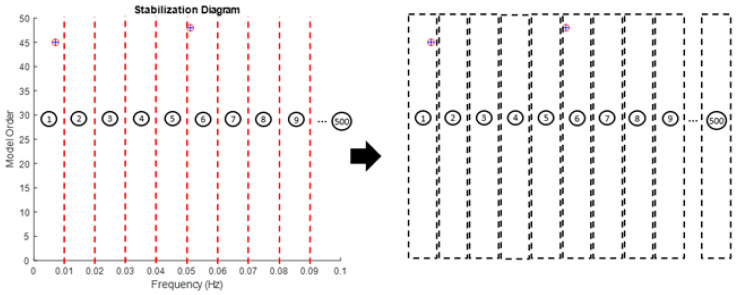
Illustration of example generated input images from a stabilization diagram with a 0.1 Hz interval frequency for feature extraction for 5 Hz maximum frequency.

(15) xlim([(p−0.01)     p])

where p is the value of natural frequency. Thus, every image represents a frequency of 0.01 Hz. The process of this procedure is shown in Figure 3 and Figure 4 below. In order to make image feature extraction more efficient, all axes and legends in the plot of these images have to be removed.

#### 2.3.2. Image Feature Extraction

Then, the standardized image features from MATLAB software (a multi-paradigm numerical computing environment and proprietary programming language developed by MathWorks) were applied in this study to extract the image features of each image of stabilization diagrams that were previously generated. These features specifically represent the characteristics of each parameter (natural frequencies, damping ratios) for different conditions, either stable or unstable. The standardized image feature, Maximally Stable External Regions based on regions as the characteristic value, was used in this study in order to capture all the modes of interest particularly in terms of computational mode appearance. 

**Figure 5:**
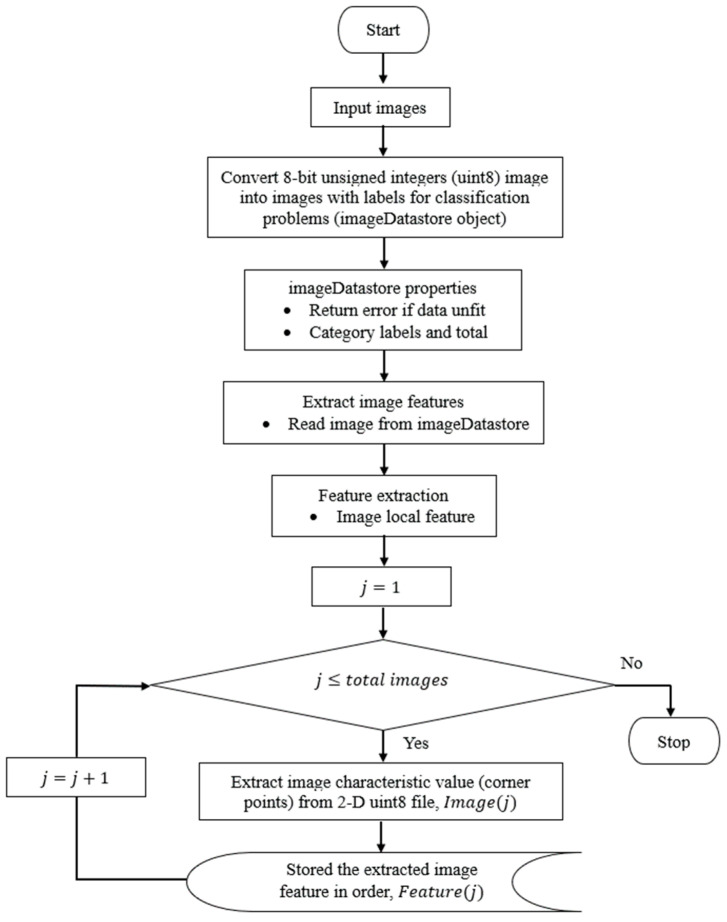
Flowchart process of image feature extraction.

Generally, the image feature will provide a certain value based on its image characteristics. If the image is blank, the value will become zero, otherwise, if the pole appears, the value will increase. The increasing value of image feature extraction depends on the number of poles appeared in that particular image. It works well with stabilization diagram because the stabilize physical modes consist of the vertical alignment of stable poles, while noise modes are scattered. By generating the image of stabilization diagram according to 0.01 interval frequency (cut down vertically), the poles can be clustered accordingly. Therefore, the generated input image based on the interval frequency of stabilization diagram plays a key role in the performance of the image feature extraction. Details explanation about the process of this procedure was shown in Figure 5 below.

**Figure 6:**
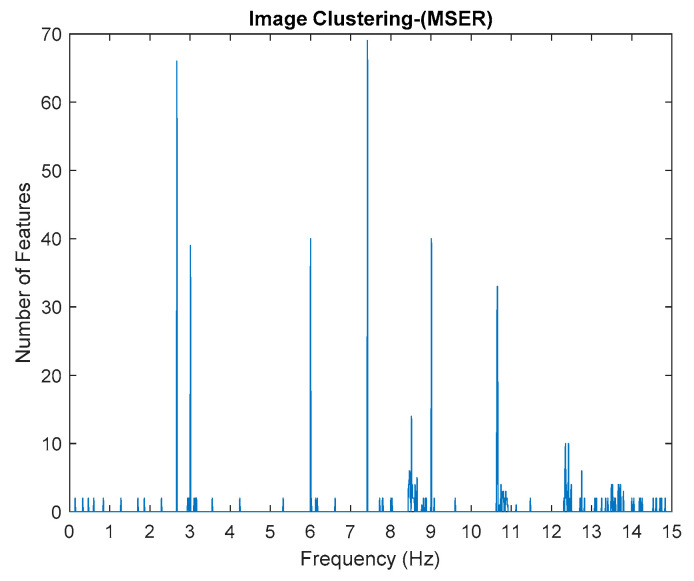
Image clustering plot by using image features extraction from Maximally Stable External Regions (MSER) of the three-story frame model from numerical simulations.

**Figure 7:**
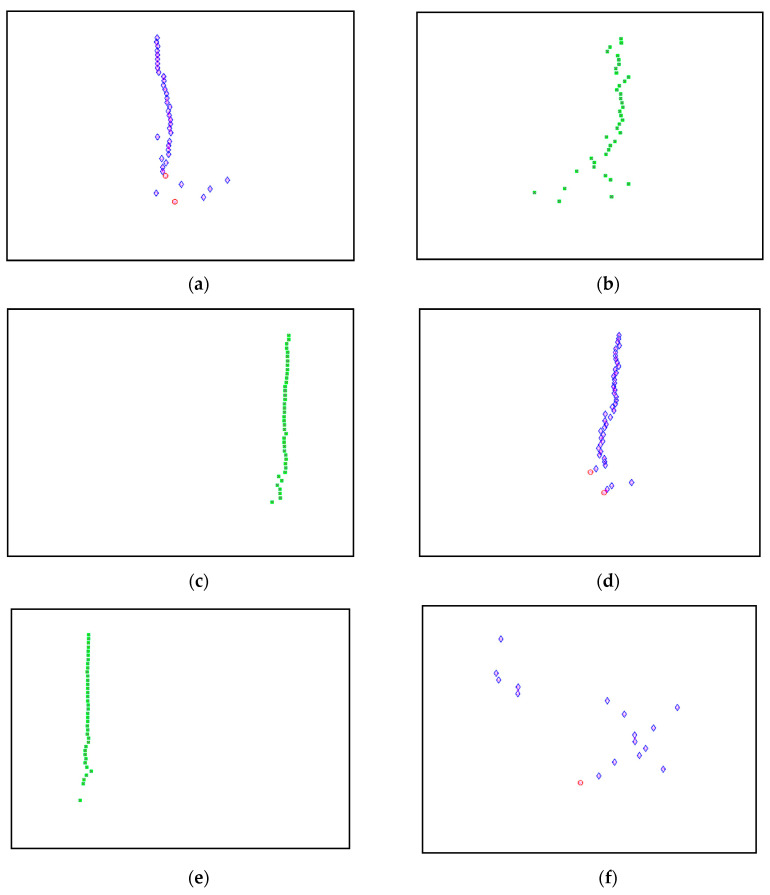
(**a**–**f**) First, second, third, fourth, fifth and sixth physical modes respectively, of the three-story frame model from numerical simulations.

This algorithm was configured to group all the physical poles of the stabilization diagram by using image features extraction and then constructing an image clustering plot, as shown in Figure 6 below. The selection of the physical modes of the system that were autonomously implemented, involved MATLAB command—*find* and the threshold in order to discriminate the weak ones and leave only the dominant modes. The approach is based on excluding the weak modes with lower value than the threshold. The threshold is set to be 20 features in an image clustering plot (10 poles only considered as stable). The example of the generated images that have been classified as physical modes from the clustering plot are displayed in Figure 7.

### 2.4. Classification of Structural Modes and Harmonic Component Using Shape Recognition

The classification of structural modes and the harmonic component of the system was autonomously implemented in MATLAB using shape recognition process. 

**Figure 8:**
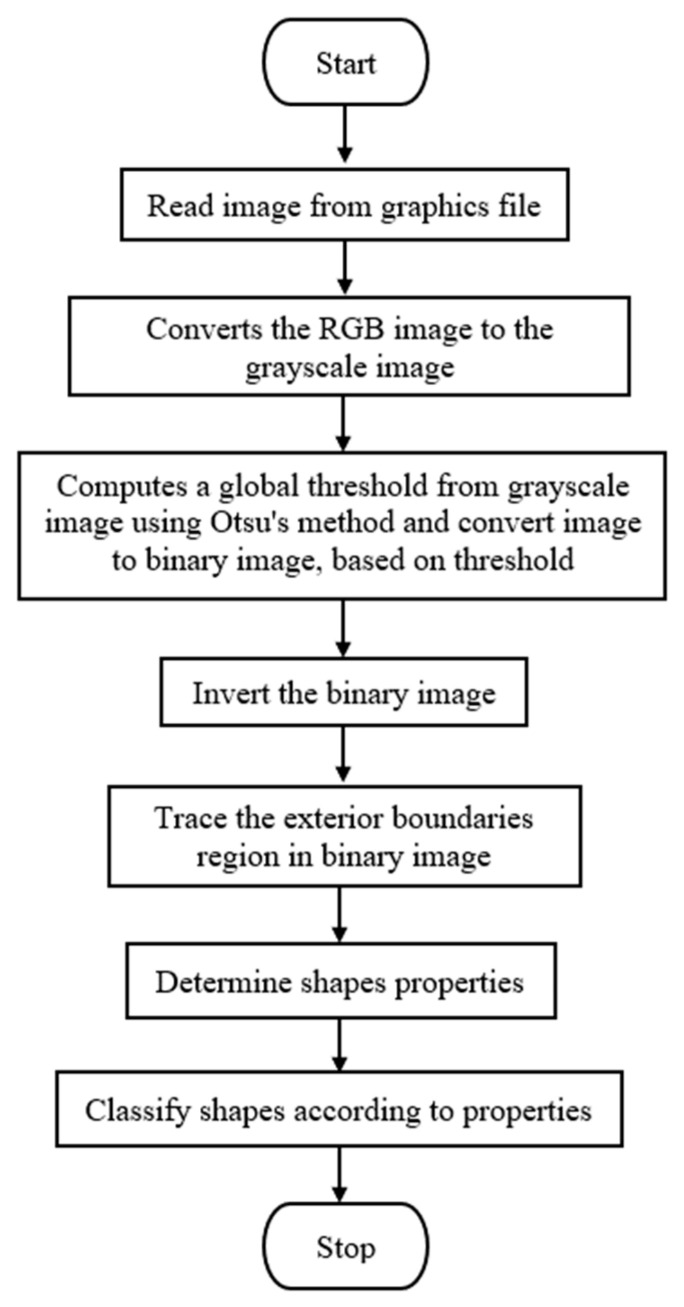
Flowchart of shape recognition and classifier steps. RGB image refers to three hues of light that can be mixed together to create different colors image.

**Figure 9:**
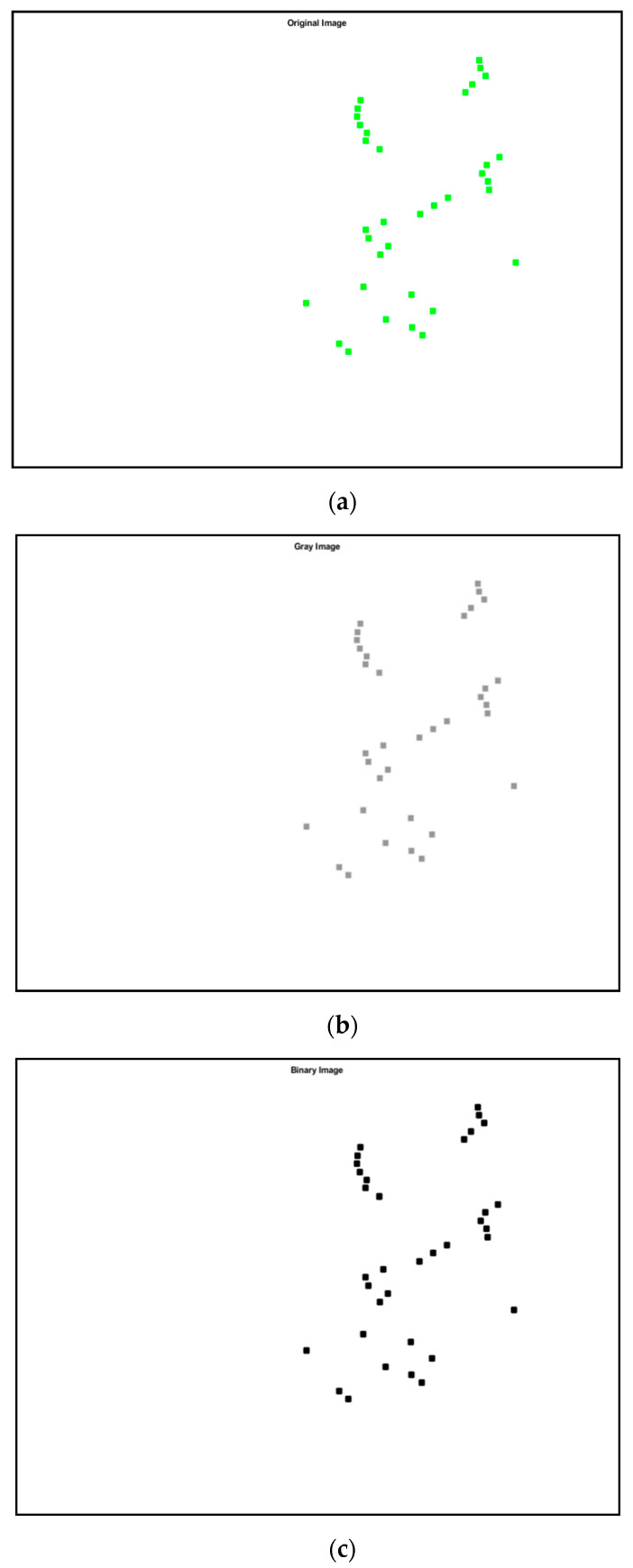

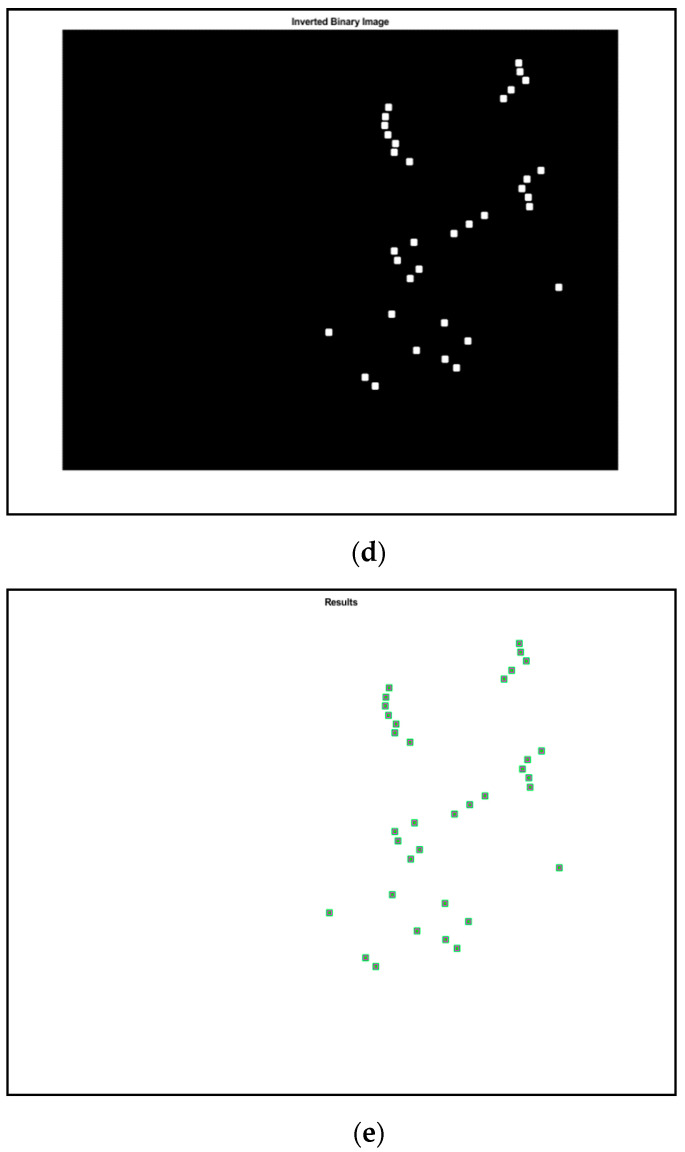
Steps of the shape recognition process (**a**) original image, (**b**) grey image, (**c**) binary image, (**d**) inverted binary image and (**e**) results in images respectively, of the three-story frame model from numerical simulations.

The process of shape recognition starts with the reading input image from the graphics file. Then, the input RGB image (stands for “Red Green Blue” and refers to three hues of light that can be mixed together to create different colors image) is converted to the greyscale image by eliminating the hue and saturation information while retaining the luminance. The computation of a global threshold from the grayscale image is performed using Otsu’s method [36] before being converted into a binary image based on this threshold by replacing all pixels in the input image with luminance greater than a level with the value 1 (white) and replacing all other pixels with the value 0 (black). Otsu’s method chooses a threshold that minimizes the intraclass variance of the threshold black and white pixels. Next, the binary image is inverted and the subsequent process of tracing the exterior boundaries region is implemented in order to determine shape properties. Lastly, all input images are classified according to shape properties that have been identified before. The overall steps of the shape recognition and classifier process is shown in Figure 8 and Figure 9.

**Figure 10:**
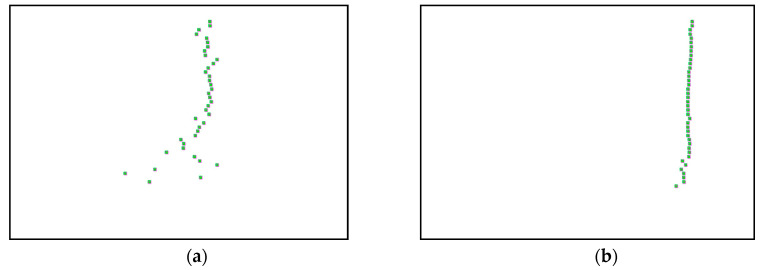

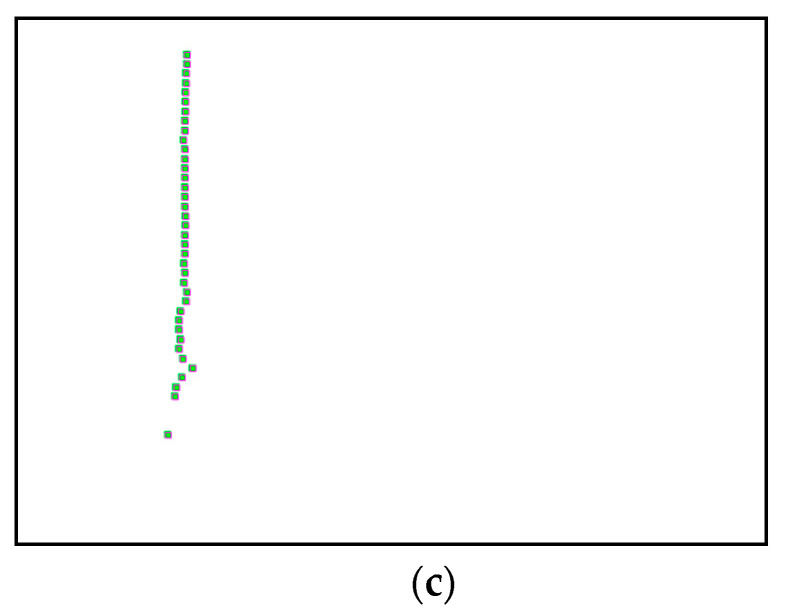
(**a**–**c**) First, second and third harmonic modes respectively, detected by magenta square marker type, of the three-story frame model from numerical simulations.

Harmonic frequencies are determined based on the presence of square-shape poles recognized inside an image that represent a harmonic component. The square-shape pole is detected by a magenta square marker type as shown in Figure 10 below and the obtained image number represents harmonic frequency after multiplying by 0.01 because every image represents the frequency of 0.01 Hz.

### 2.5. Remove Harmonic Signal Using Sinusoidal Model Fitting

The next step is to remove the harmonic signal by applying nonlinear regression (by the use of MATLAB command, *nlinfit*) of the responses input signal on the predictors in time using a sinusoidal model function, $y_{s}$, as in Equation (16) below, since harmonic excitations produce a deterministic sinusoidal response at their excitation frequency throughout the measurement duration [19]:

(16) $$y_{s}=A\sin\left(2\pi tB\right)$$

where t represents the vector of time, A and B are the coefficients parameters to be fitted. The coefficients are estimated using iterative least squares estimation, with initial values specified by [A B]. The value of A and B are 1 and the identified harmonic frequencies (obtained from the previous step) respectively.

Then, the estimated regression coefficients are used to reconstruct the sinusoidal signal that represents harmonic frequency. Then, the generated sinusoidal signals, $y_{h}$ are automatically removed from the original signal, $y_{o}$ and leave only the useful input time series response signal, $y_{c}$ that free from the harmonic component by simple means, as shown in Equation (17):

(17) $$y_{c}=y_{o}-y_{h}$$

### 2.6. Estimate Modal Parameter

**Figure 11:**
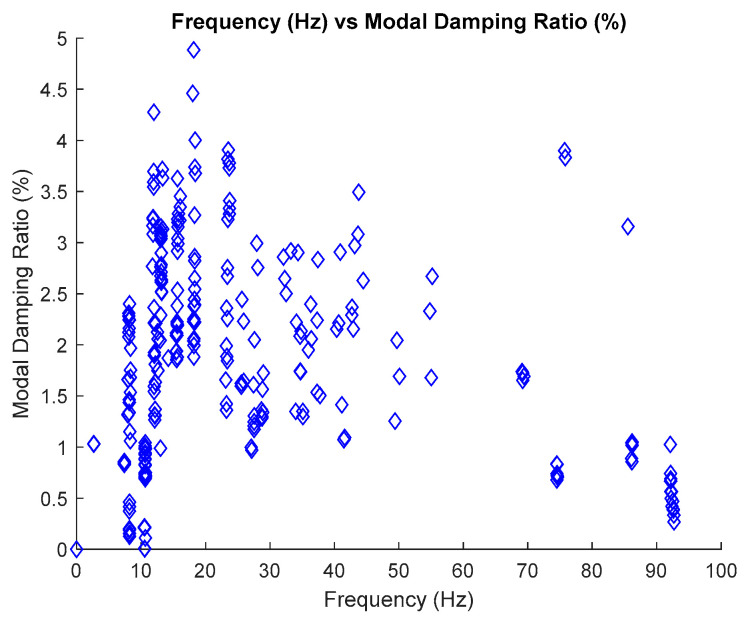
Plot frequency versus modal damping ratio of the three-story frame model from numerical simulations.

The identification of modal parameters is essential in order to determine the inherent dynamic characteristics of a system. For the estimation of natural frequencies, it has been discussed in the previous section. Therefore, this section only focuses on the estimation of modal damping ratios. Generally, the process of estimating the modal damping ratios depends on the identified natural frequencies which are based on the frequency versus modal damping ratios plot in the stabilization diagram. Only stable poles are chosen to be displayed in this plot, as shown in Figure 11 below.

The process of extracting modal damping ratios from the frequency versus modal damping ratio plot involves the same process as the natural frequency, which uses image clustering, but in terms of the generated input images, it is quite different. 

The process of the generated input image for modal damping ratios from the frequency versus modal damping ratios plot requires the identified natural frequencies as a fixed x-axis and the value of modal damping ratios along the y-axis. In this case, the input image was generated and displayed separately into every value of the modal damping ratio according to a 0.002 interval along the y-axis with the fixed value of identified natural frequency in the x-axis, as characterized in Equations (18) and (19), respectively. In order to make image feature extraction more efficient, all axes and legends in the plot of these images must be removed.

(18) ylim([(p−0.002)     p])

(19) xlim([(d−0.01)     (d+0.01)])

where p and d are the value of modal damping ratio and estimated natural frequency respectively, in the frequency versus modal damping ratio plot. The selection of 0.002 as an interval for the generated modal damping ratio along the y-axis is because it can provide more sensitivity and more accurate results. The results of the estimation modal damping ratio according to the 0.002 interval exhibits very satisfying results that are close to target value compared to the 0.01, 0.005 and 0.001 intervals. The use of the 0.002 interval is appropriate to capture all the poles of interest. The process is repeated for each mode because each mode has its own natural frequency. In order to make image feature extraction more efficient, all axes and legends in the plot of these images must be removed.

The standardized image features from MATLAB, Maximally Stable External Regions based on regions as the characteristic value, was applied to extract the image features of each image of stabilization diagrams that were previously generated. The image feature will provide a certain value based on its image characteristics. If the image is blank, the value will become zero, otherwise, if the pole appears, the value will increase. Each pole presents the value of 2, the increasing value of image feature extraction depends on the number of poles that appeared in that particular image. This identified image features extraction are then constructed in image clustering plot, as shown in Figure 21. The process is repeated for each mode because each mode has its own natural frequency. 

The estimation of the modal damping ratio for each mode is autonomously implemented, and involves the MATLAB command—*find* and is based on the maximum number of images featured in image clustering plot. 

## 3. Results and Discussion

### 3.1. Validation with Numerical Simulations

**Figure 12:**
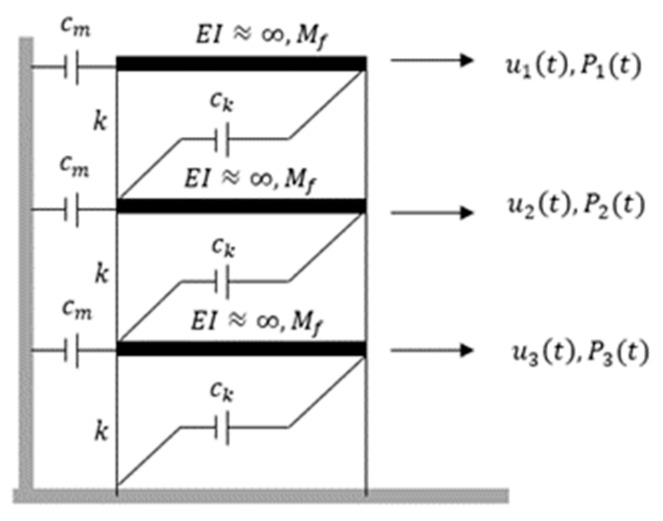
Three-story frame models.

As a fundamentally necessary condition, the algorithm has been assessed first from computed numerical responses based on random white noise, acting on different ideal shear-type frame structures. In this section, the proposed algorithms are validated using simulated data from a simple three-story shear-type model consisting of three degrees of freedom (DOF), as illustrated in Figure 12. All these structural features with different DOFs are examined first [37,38,39]. 

The characteristics of the simulated three-story shear-type models which present well-separated modes are provided in Appendix A by the stiffness (K), mass (M) and damping (C) matrices and by the corresponding modal parameters: natural frequencies, modal damping ratios and mode shapes. The well-separated modes and different DOFs are used in this study in order to discover the effectiveness of the proposed approach for the diversity of features and type of structure. The damping of the structure is viscous (damping forces proportional to velocity) and proportional Rayleigh damping, described by mass and stiffness:

(20) $$\left[C\right]=\left[C_{m}\right]\;+\;\left[C_{k}\right]=a_{0}\left[M\right]+a_{1}\left[K\right]$$

where $\left[C_{m}\right]$, $\left[C_{k}\right]$, [M] and [K] are the mass proportional damping, stiffness proportional damping, mass and stiffness matrices, respectively. Meanwhile, $a_{0}$ and $a_{1}$ are the coefficients describing the mass and stiffness proportional damping. 

To determine the coefficients $a_{0}$ and $a_{1}$, the prescribed modal damping ratios for three-storey shear-type models are set to $\zeta_{k}$ = $\zeta_{j}$ = 1% of critical damping. In the literature, only lightly damped structures that contain modal damping ratios below 2% are considered for analysis [40,41,42,43,44,45]. The system matrices were defined with the aim of achieving modal parameters with values of the same order of magnitude as the ones usually found in current civil engineering structures.

Input is taken as a stationary broadband ambient excitation with normally distributed random numbers assuming independent inputs for all DOF of the models. It has a constant power spectral density (PSD) which can cover a wide range of frequencies and is adequate to excite all the structural modes. The random input excitation, which is also known as zero-mean Gaussian white noise, takes the assumption of the excitation system to be linear and time-invariant. The response of the system is simulated using Newmark’s method with constant average acceleration (i.e., *γ* = 12 and *β* = 14) [46]. The adopted parameters in the processing are shown in Table 3 below.

The simulated outputs, which are time series with the accelerations of all the DOFs of the models, are corrupted with noise that mimics the influence of the sensors and measuring chain noise. This is simulated by normally distributed random numbers with a standard deviation equal to 10% of the standard deviation of the simulated outputs (this percentage of noise is quite conservative in the case of well-conducted ambient vibration tests [34]).

**Figure 13:**
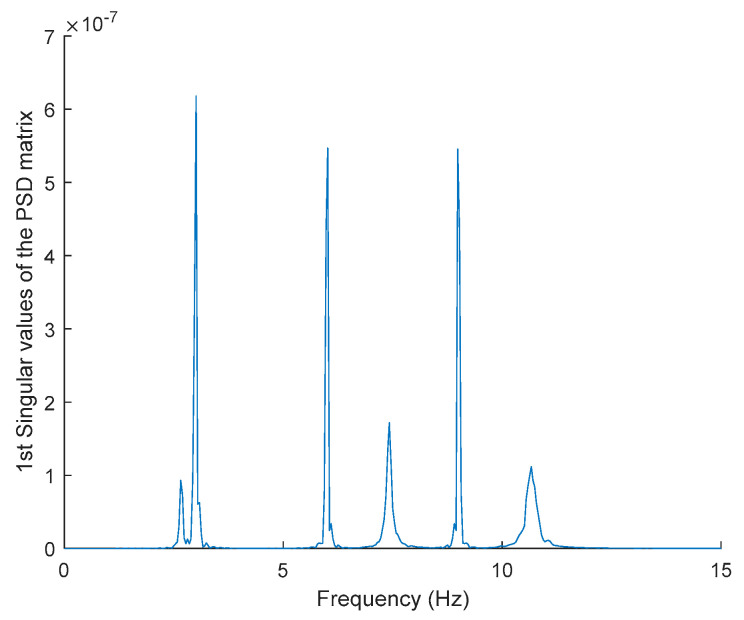
The first singular values plot of the power spectral density (PSD) matrix of the original response signal.

**Figure 14:**
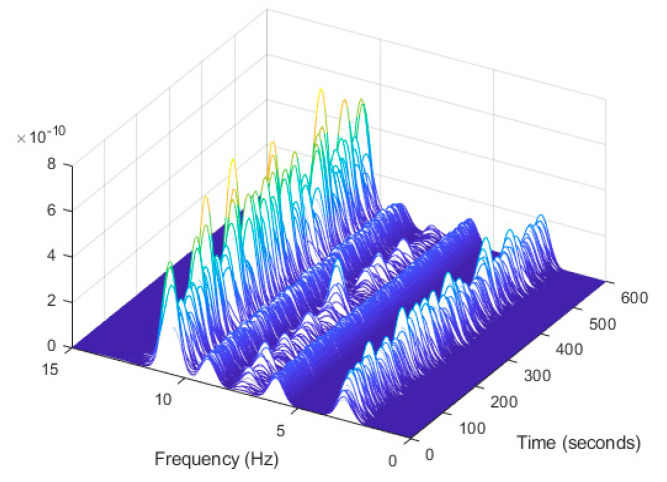
Computation the spectrogram of the original response signal displayed as a waterfall plot.

In order to evaluate the influence of the presence of harmonic components on the exciting force, and to check the efficiency of the proposed automated harmonic signal removal, a combination of harmonic excitation and Gaussian white noise excitation with zero mean is applied to each mass separately. The frequencies of the applied harmonic excitation to structural modes are equal to 3 Hz and multiples of it (6 Hz and 9 Hz), as shown in Table 4. The estimated first singular value of the power spectral density (PSD) matrix and the spectrogram of the simulated temporal response are shown in Figure 13 and Figure 14 respectively, which present six dominant peaks combining structural and harmonic modes. Among the six dominant modes, three of them are harmonic component frequencies and the other three are structural modes. The aim of this study is to determine the effectiveness of the proposed approach in the case of the closely spaced modes between harmonic and structural modes.

The effectiveness of the proposed algorithm is verified by the target values of all the structural modal parameters on three-story frames using eigenvalue problem analysis and the assigned value for the modal damping ratio, as described in Table 5 below. In order to describe the robustness of the proposed algorithm, the numerical simulation was run.

**Figure 15:**
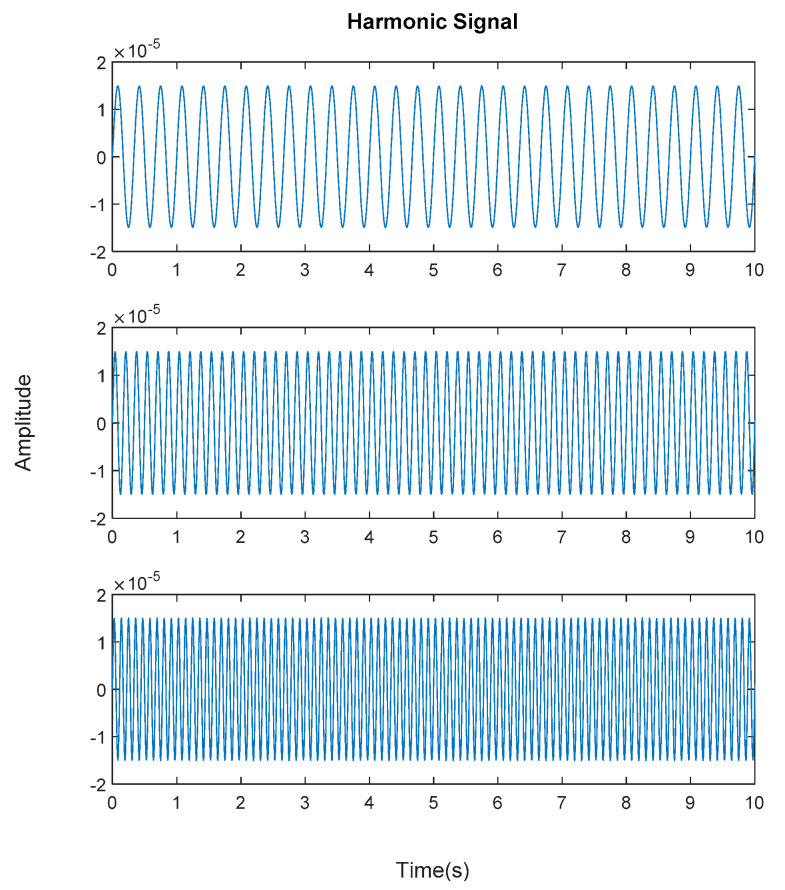
Generated harmonic signal based on estimated coefficients using iterative least squares estimation. Top figure is the first harmonic component frequency; middle is second harmonic component frequency and below is the third harmonic component frequency in time domain.

**Figure 16:**
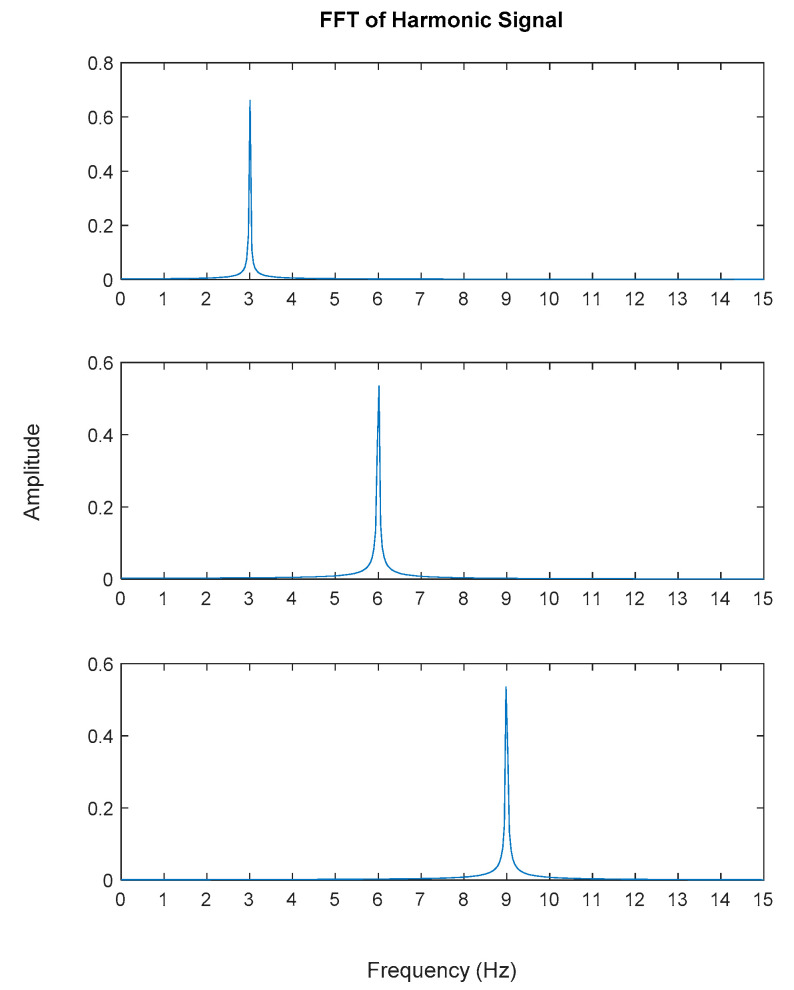
Fast Fourier Transform (FFT) of generated harmonic signal based on estimated coefficients using iterative least squares estimation: Top figure is the first harmonic component frequency; middle is second harmonic component frequency and below is the third harmonic component frequency in frequency domain.

**Figure 17:**
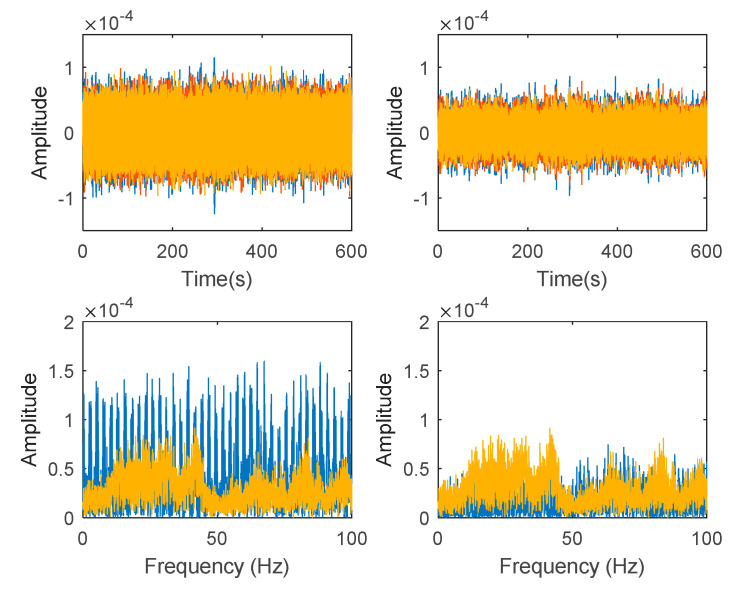
Top and bottom plots are in the time domain and frequency domain respectively, while left and right side indicate signal before and after the harmonic removal process.

**Figure 18:**
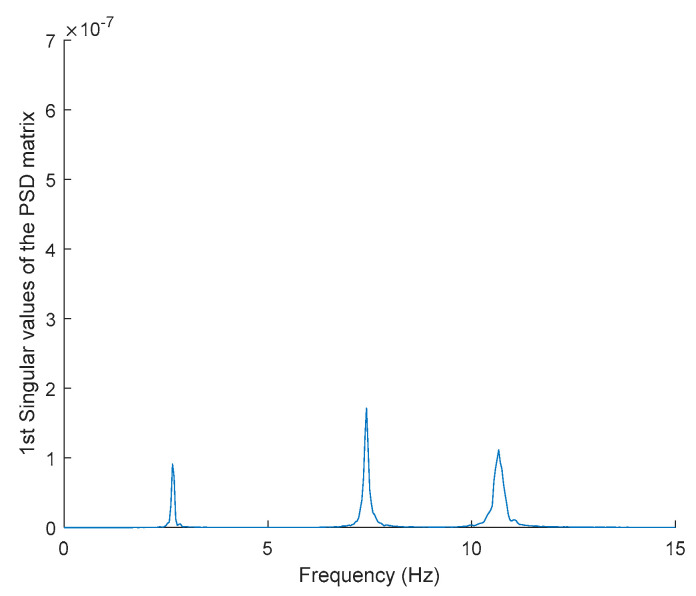
The first singular values plot of the PSD matrix of the response signal after removing harmonic components.

**Figure 19:**
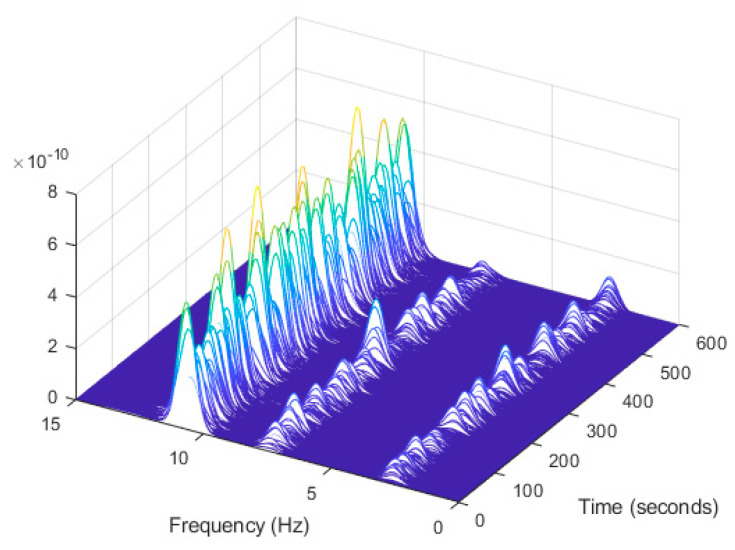
Computation of the spectrogram of the denoising harmonic signal displayed as a waterfall plot.

**Figure 20:**
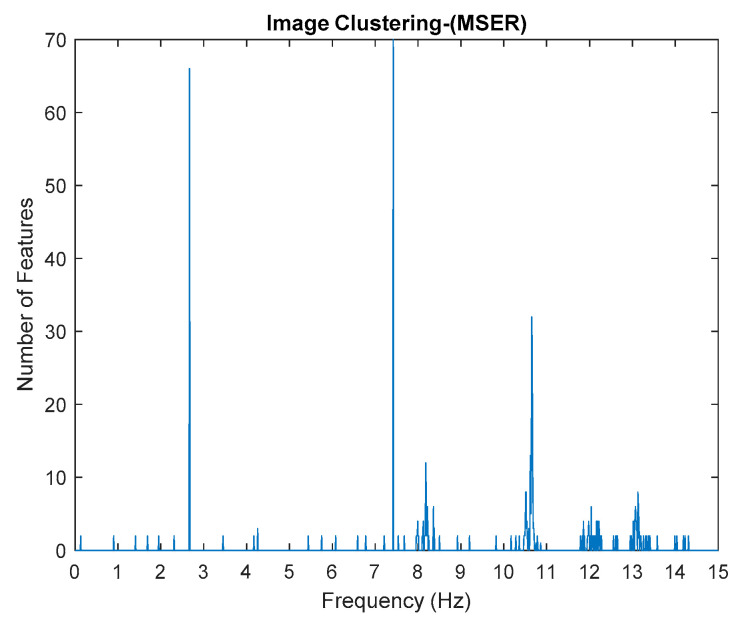
Image clustering plot by using image features extraction from Maximally Stable External Regions after removing harmonic components.

The results of the automated harmonic removal technique are characterized in Figure 15, Figure 16, Figure 17, Figure 18, Figure 19 and Figure 20 and Table 6, Table 7 and Table 8. 

Generated harmonic signals in the time and frequency domain are shown in Figure 15 and Figure 16, respectively.

**Figure 21:**
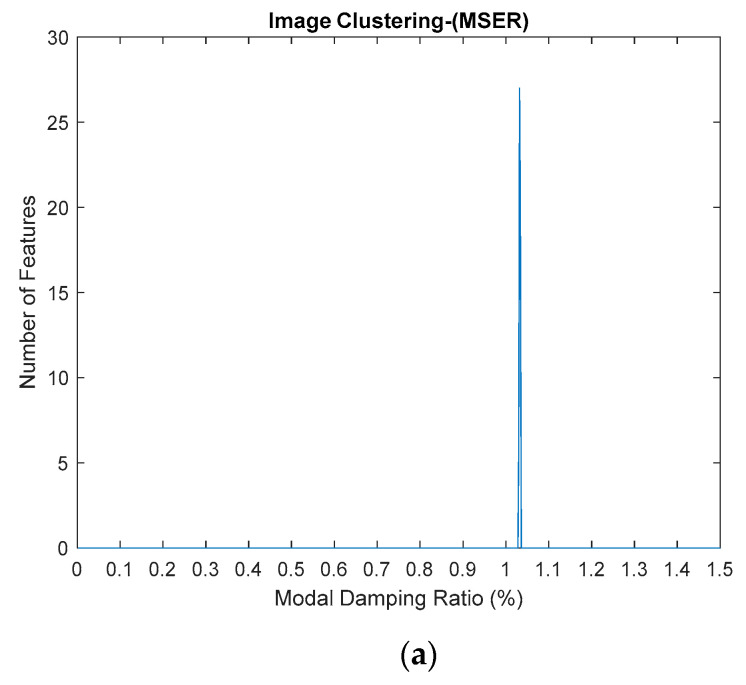

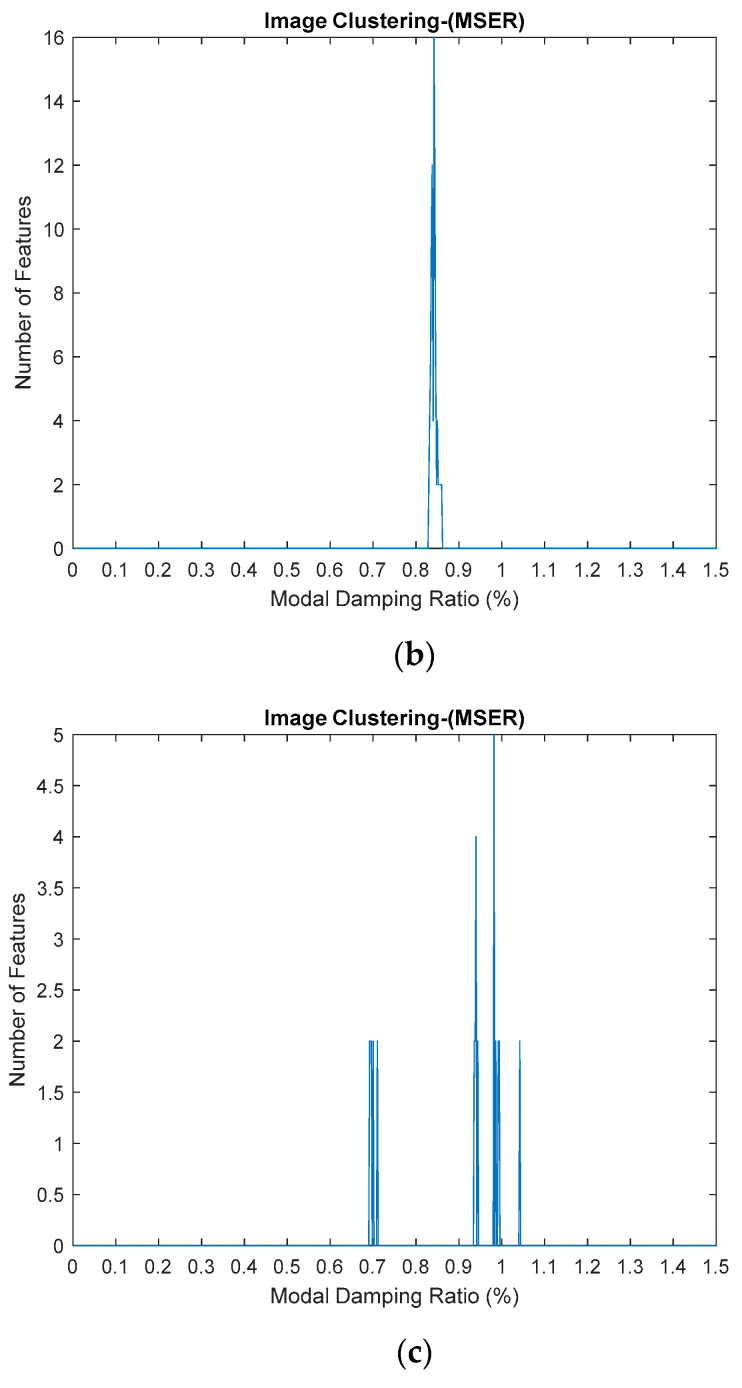
(**a**–**c**) First, second and third modes for modal damping ratios respectively, of the three-story frame model from numerical simulations by using the standardized image feature, Maximally Stable External Regions (MSER) based on regions as the characteristic value.

The image clustering plots for identifying modal damping ratios for the three-story frame model from numerical simulations after removing the harmonic signal are displayed in Table 9 and Figure 21 below.

Results obtained from the two different approaches are also presented in Table 10, Table 11, Table 12 and Table 13 below and compared with the proposed SSI-Covariance with the image-based feature extraction technique.

Based on the image clustering result, plotted by using image features extraction from Maximally Stable External Regions as displayed in Figure 6 with all the poles of the stabilization diagram that are presented in Figure 2. Stabilization diagrams were used as an indicator to distinguish the physical poles from the spurious modes which also consists of harmonic components by adjusting the limit values of modal damping ratios. Using standardized image features in MATLAB, image clustering able to capture and provide a clear appearance of all modes of interest including structural modes and harmonic components in the stabilization diagram is characterized in Table 6. This standardized image feature plays a vital role in identifying which image represents the vertical alignment of stable modes. Clear identification of the harmonic component mode is at the frequency of 3, 6 and 9 Hz by using image shape recognition that successfully classifies the harmonic components from structural modes whose results are shown in Table 7. Knowing the frequency that represents the noise mode or the harmonic component is essential because it can be used for the next step to reconstruct a sinusoidal signal that represents harmonic frequency by using a sinusoidal model fitting, of which results are displayed in Figure 15 and Figure 16. Then, the generated sinusoidal signals are automatically removed from the original signal to become a clean input time-series signal that frees from the harmonic component. The results of estimated coefficient values for the sinusoidal model fitting of harmonic components in Table 8 exhibits very accurate results with respect to the target values. The results after going through the harmonic removal process can be clearly seen in Figure 17, Figure 18, Figure 19 and Figure 20, where only structural poles are left and are free from harmonic components poles.

In order to demonstrate the novel ability of the proposed SSI-Covariance with image-based feature extraction technique to eliminate harmonics, modal parameters are also identified using the SSI method, classical SSI-Covariance and SSI-Data. Results obtained from the two different approaches are also presented in Table 10, Table 11, Table 12 and Table 13 and compared with the proposed SSI-Covariance with the image-based feature extraction technique. Table 10 and Table 11 provide the results of estimated natural frequency before and after removing the harmonic components, respectively. Meanwhile, Table 12 and Table 13 indicate the results of estimated modal damping ratios for before and after removing the harmonic components, respectively. Detailed results of modal damping ratios displayed in the image clustering plot for each mode are also presented in Figure 21 which have been generated from the frequency versus modal damping ratio plot in Figure 11.

Results of the proposed automated SSI-Covariance with a novel approach using image-based feature extraction in estimating modal parameters, particularly natural frequencies, demonstrated very high accuracy, exhibiting an average percentage deviation below 1% for all cases. In addition, results of the estimated modal damping ratio are quite consistent and provide limited errors with percentage deviation (error) below 4%, where classical SSI-Covariance can be up to 30% of percentage deviation (error) before removing harmonic components from the response signal as presented in Table 12. On the other hand, results of estimated modal parameters using SSI-Data are also quite consistent and with limited errors with a percentage deviation (error) below 5% for modal damping ratios. Meanwhile, the results of the estimated modal damping ratios after removing the harmonic components from the original response signal as in Table 13 showed very limited errors and improved significantly compared to before removing the harmonic components for all approaches. For the proposed approach, results of percentage deviation (error) with respect to target values are below 3.2%, where classical SSI-Covariance can be reduced to around 4.5% of percentage deviation (error) and SSI-Data with percentage deviation (error) also below 3.2%. This shows that the corrupted response signal with unwanted mode not only affects the decision making for the actual modes selection but also strongly influences the identification of modal damping ratios by the presence of harmonics.

By comparing the results of the estimated modal parameter using the proposed automated harmonic signal removal using SSI-Covariance with image-based feature extraction with other SSI methods, which are classical SSI-Covariance and SSI-Data, the results yield quite accurate and consistent results for before and after removing the harmonic component and it seems not very affected with corrupted response signal with unwanted mode. Moreover, this approach is also highly efficient in identifying and clearly separating closely spaced modes, as seen in Figure 6 for the first two closely spaced modes from 2.6 to 3 Hz.

The results of this study also prove that the implementation of image-based feature extraction for identification and classification of structural modes and harmonic component in a stabilization diagram and sinusoidal model fitting as a tool for filtering purposes are a good combination as well as an effective procedure, producing a good input signal that is free from unwanted modes.

### 3.2. Validation with Experimental Testing

**Figure 22:**
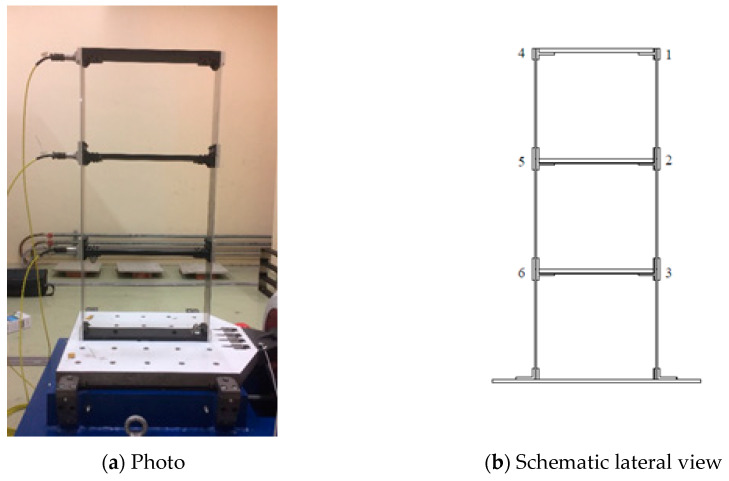
General view of the metallic frame.

A three-story steel frame is set up to experimentally demonstrate the applicability of the proposed approach and to illustrate the theoretical concepts, described in this chapter. The masses on each story are dominant and it is composed of 3 rigid iron masses connected to each other and to the base through flexible aluminum columns by connection elements, which are clamped at nodes 1–6 and the base (Figure 22). Horizontal impact at the top floor is applied to the system, and the responses are measured and transmitted into the data acquisition channels and then to the computer. The proposed algorithm is implemented on the measured data.

In order to illustrate the complex candidate models of OMA, a vibration experiment of the metallic frame was performed in the laboratory. It is assumed that the lateral response on nodes 1 and 4, 2 and 5, as well as on 3 and 6, are equal. So, only acceleration signals in nodes 1, 2 and 3 were measured.

**Figure 23:**
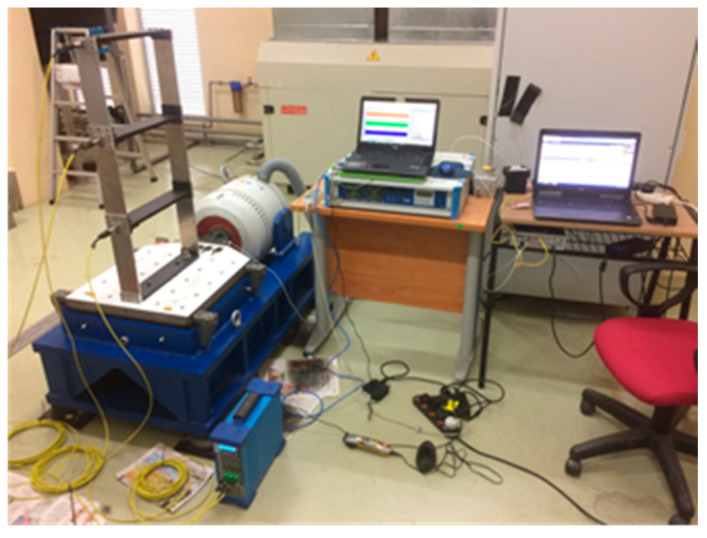
Instruments set up.

The signal acquisition system used in the experiment consists of wicoson accelerometers, signal conditioners and data acquisition equipment, as shown in Figure 23. The acceleration signals were measured with sensors that are attached on top of each mass to record the system responses and amplified with an injection control pressure (ICP) sensor signal conditioner. The signals were sampled by OROS data acquisition equipment (DAQ) which is connected to a computer and control and using NVGate software. The frame was excited by the slip shaker in order to induce random excitation.

In order to induce random input excitation, a slip shaker is used to excite the three-story steel frame. It has a constant PSD which can cover a wide range of frequencies and is adequate to excite all the structural modes [21]. The random input excitation, which is also known as zero-mean Gaussian white noise, takes the assumption of the excitation system to be linear and time-invariant, which is characterized by constant parameters if all its fundamental properties are invariant with respect to time.

**Figure 24:**
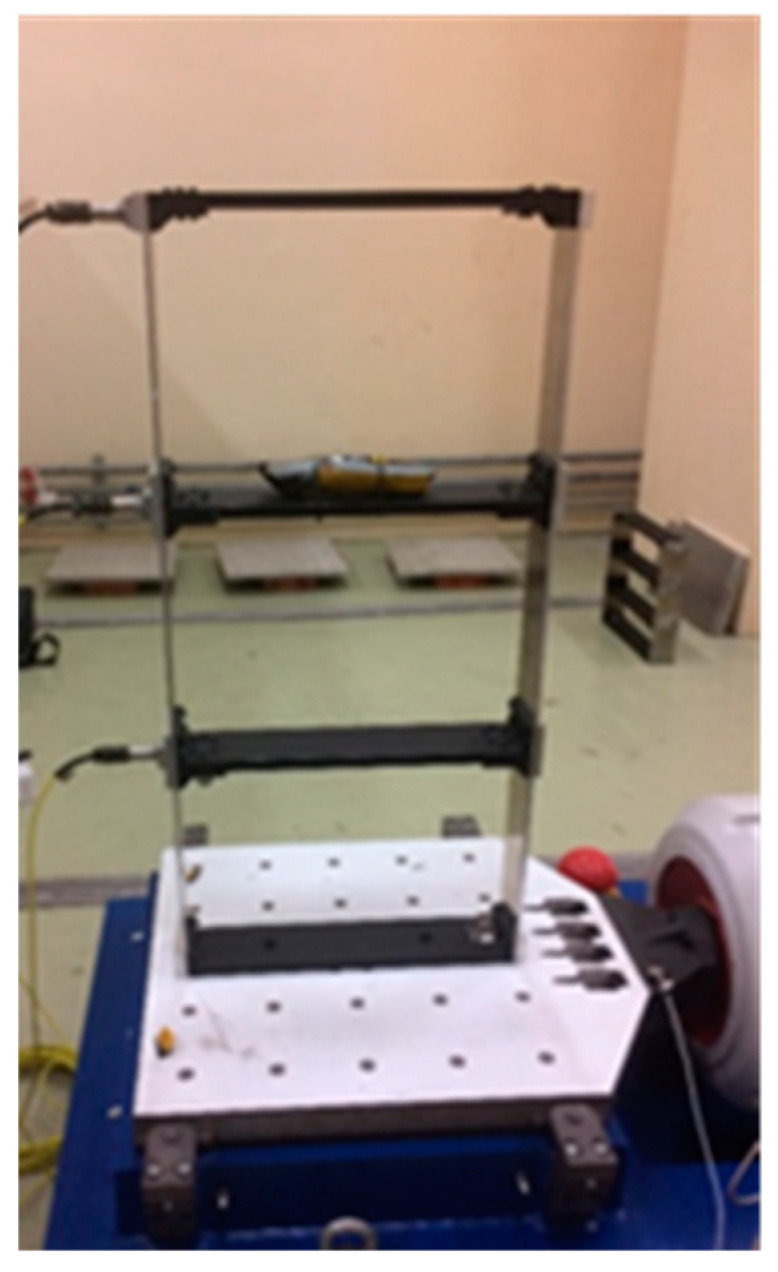
Placement of rechargeable hair trimmer on the second floor.

In addition to the random input excitation, the three-story steel frame is excited by harmonic excitations provided by a motor of rechargeable hair trimmer turning at a nominal rotation of 9000 revolutions per minute (rpm), peaks at harmonic excitation of 150 Hz and multiples of it are expected for top power motor with the shaft connecting the motor and blade. It has been placed on the second floor of the three-story steel frame, as shown in Figure 24. Thus, only the measured response at the second floor was used for the analysis. The handle of a rechargeable hair trimmer is coated with adhesive tape. The coating is needed to attenuate the excitation because otherwise, excitation levels would exceed the measurement range of 7 g of the accelerometers.

The duration of each setup was 5 min and the maximum frequency was 250 Hz. The DAQ employs some fixed signal processing filters which need to be addressed when taking measurements. The hardware does not actually sample input channels with the specified sample rate frequency sampling (fs) but samples it with 128 times the specified sample rate. This is to prevent aliasing effects. A sampled dataset can only represent a limited bandwidth of the input signal. It can only distinguish signal components with a maximum frequency of $f_{Ny}=f_{s}/2.56$, called the Nyquist frequency. If the analog input signal contains frequency components higher than the Nyquist frequency, the sampler modulates those frequency components back into the baseband from 0 Hz to $f_{s}/2.56$. This is called aliasing. The adopted parameters in the processing are shown in Table 14 below.

**Figure 25:**
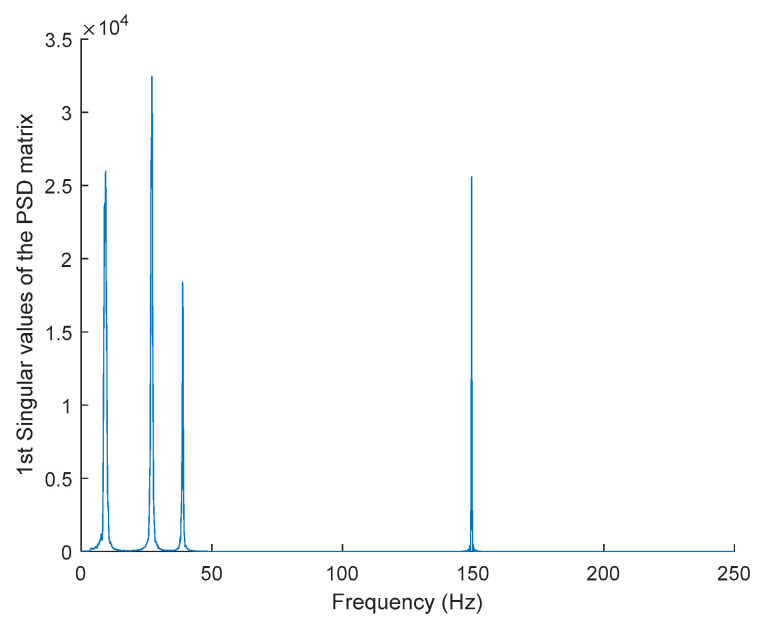
The first singular values plot of the PSD matrix of the original response signal.

**Figure 26:**
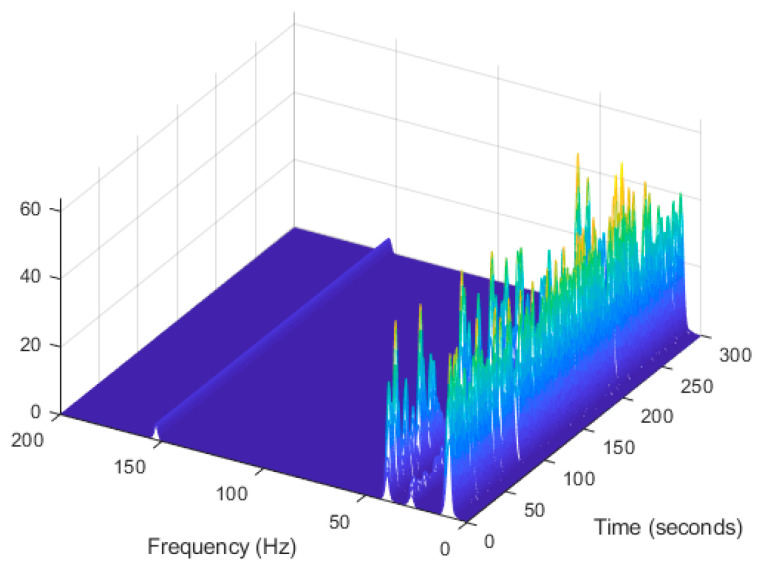
Computation of the spectrogram of the original signal displayed as a waterfall plot.

The estimated first singular value of the power spectral density (PSD) matrix and the spectrogram of the experimental temporal response are shown in Figure 25 and Figure 26 respectively, which present four dominant peaks combining structural and harmonic modes.

The results of the automated harmonic removal technique are characterized in Figure 27, Figure 28, Figure 29, Figure 30, Figure 31, Figure 32, Figure 33, Figure 34 and Figure 35 and Table 15 and Table 16. Meanwhile, results for modal parameter estimates consisting of natural frequencies and modal damping ratios are shown in Figure 36 and Figure 37 and Table 17, Table 18, Table 19, Table 20 and Table 21.

The proposed algorithm was analyzed under a controlled room that was carried out for experimental testing on a steel frame. The results are again consistent, even with the presence of harmonic excitation or noise mode.

**Figure 27:**
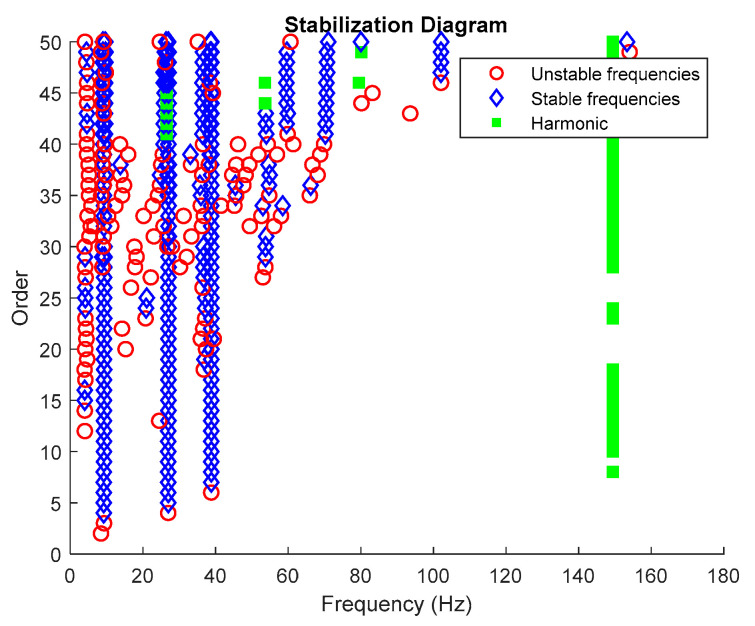
Stabilization diagram plot.

From the stabilization diagram plot in Figure 27 obtained using the proposed algorithm, we see a clear distinction between harmonics and structural poles. The blue diamond shape indicates that a pole is stable with respect to damping ratio and natural frequency and the red circle shape indicates that a pole is unstable with respect to damping ratio and/or natural frequency. Meanwhile, the green square shape indicates that a pole is a harmonic component with respect to the assigned limit values of modal damping ratios. Here, it is clear that the three stable poles at frequencies 9.27, 27.05 and 38.84 Hz are real structural poles and that the other peak is a harmonic component at frequency 150 Hz, whose results are shown in Table 15 and Table 16, respectively. In this case, only one harmonic component is considered in this analysis due to the high frequencies for multiple harmonic components. The maximum frequency of 250 Hz is more than enough to prove the efficiency of the proposed algorithm with the appearance of a harmonic component in the response signal.

**Figure 28:**
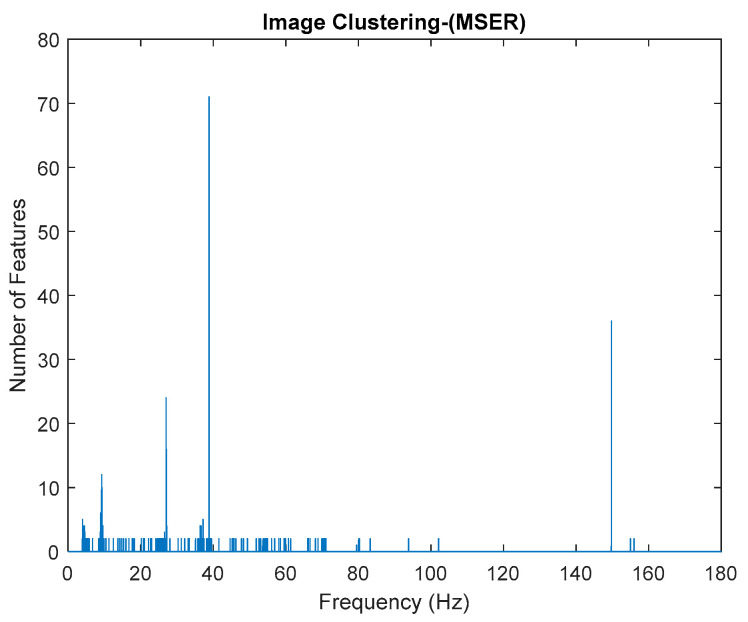
Image clustering plot by using image features extraction from Maximally Stable External Regions.

**Figure 29:**
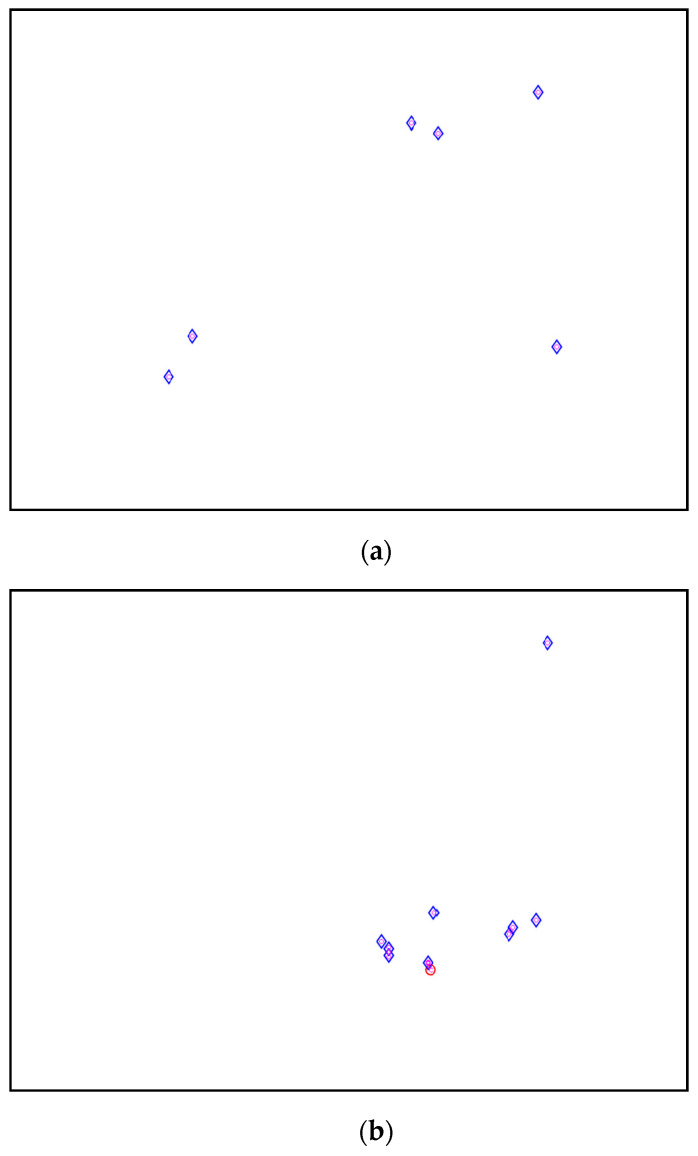

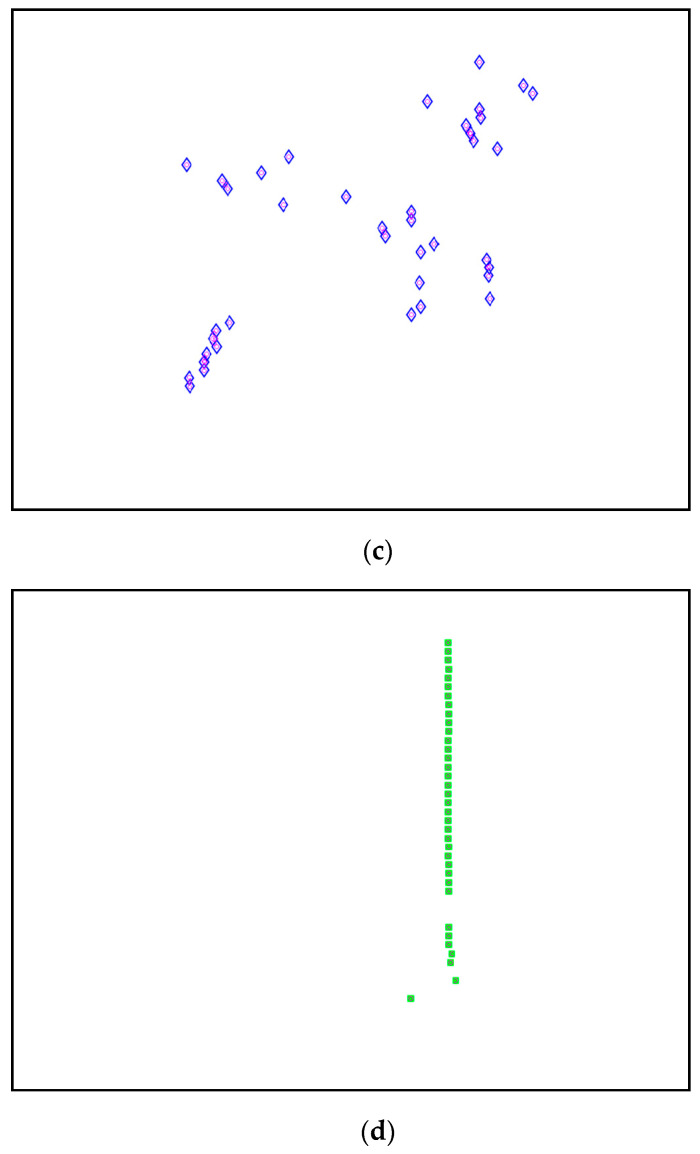
(**a**–**d**) First, second, third and fourth physical modes, respectively.

**Figure 30:**
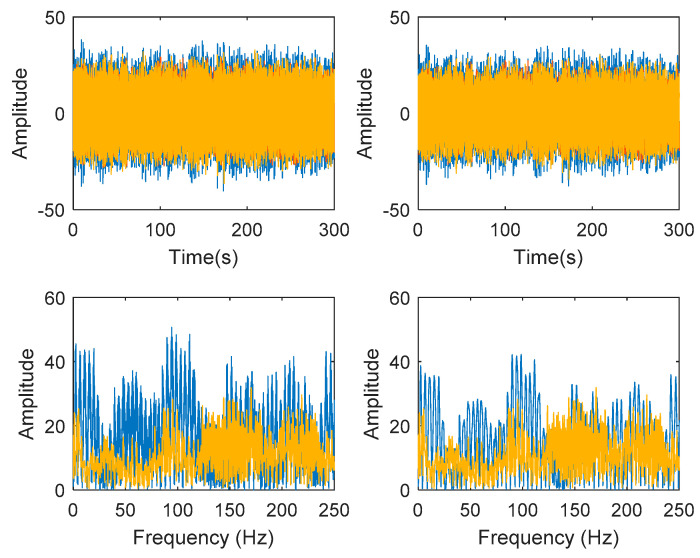
Top and bottom plots are in the time domain and frequency domain respectively, while left and right side indicate signal before and after the harmonic removal process.

**Figure 31:**
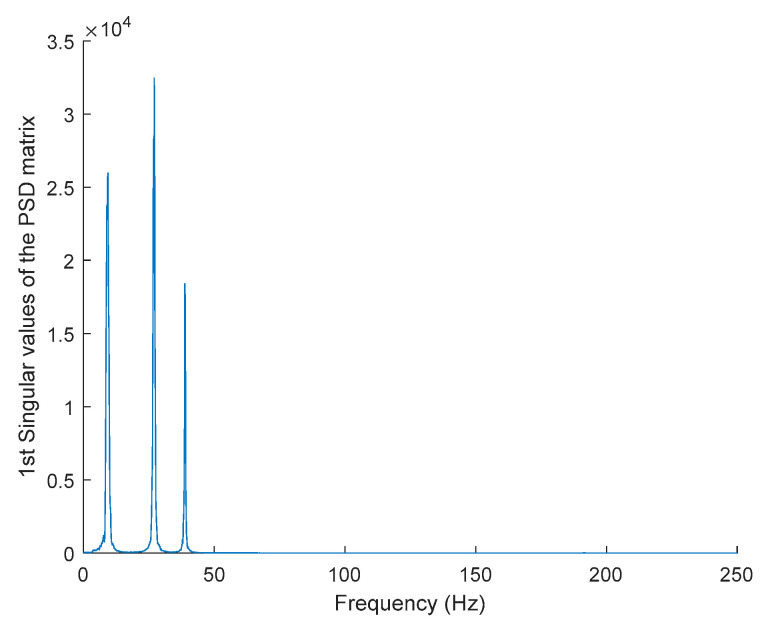
The first singular values plot of the PSD matrix of the response signal after removing harmonic components.

**Figure 32:**
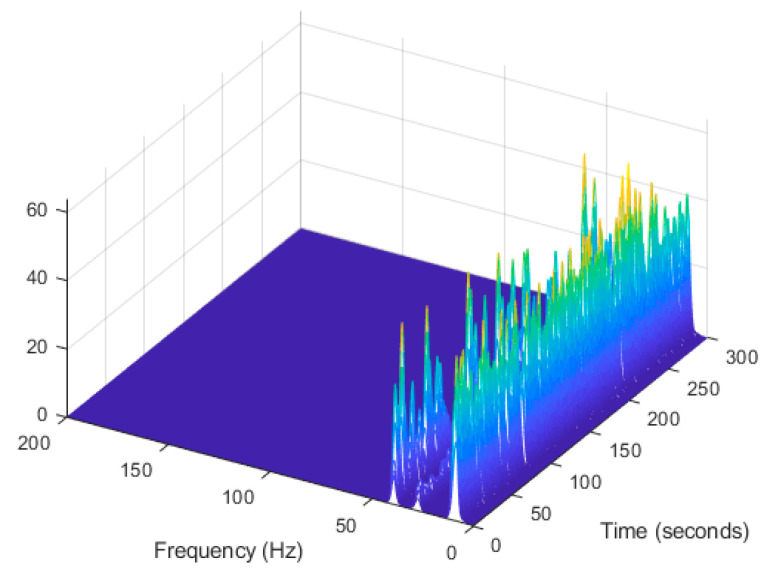
Computation of the spectrogram of the denoising harmonic signal displayed as a waterfall plot.

**Figure 33:**
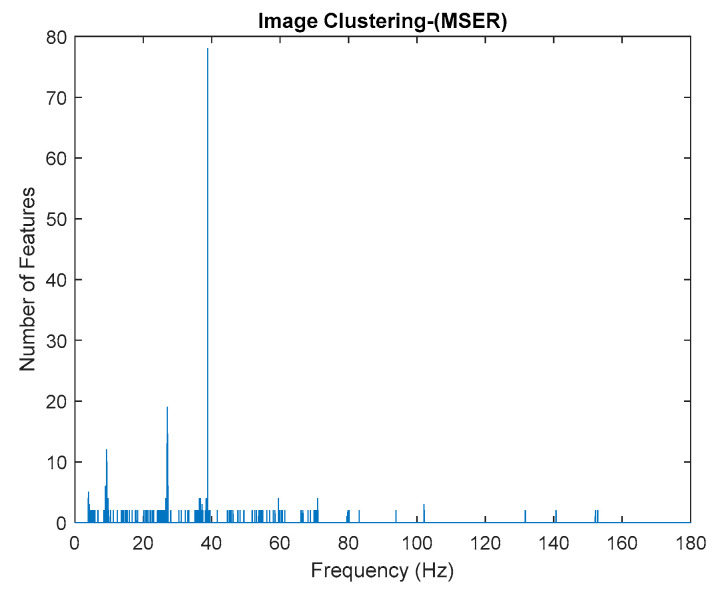
Image clustering plot by using image features extraction from Maximally Stable External Regions after removing harmonic components.

**Figure 34:**
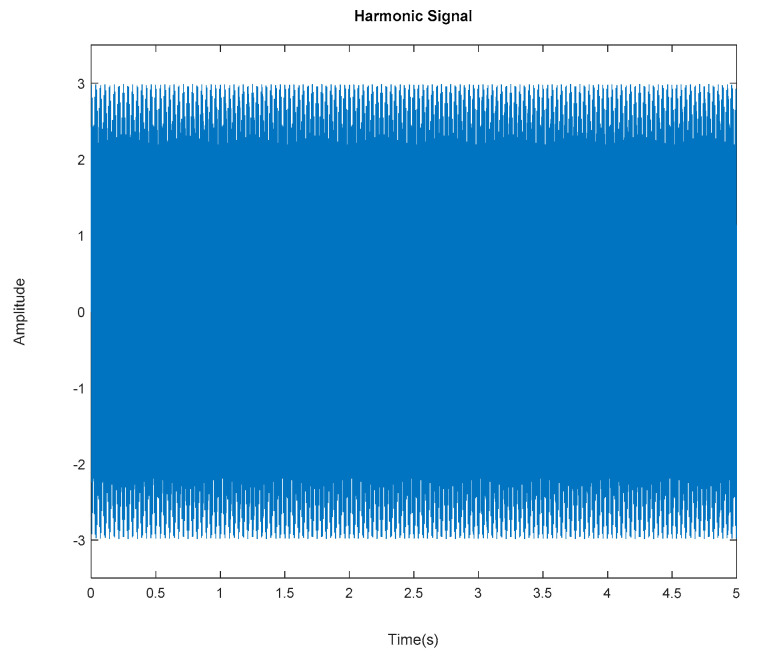
Generated harmonic signal based on estimated coefficients using iterative least-squares estimation.

**Figure 35:**
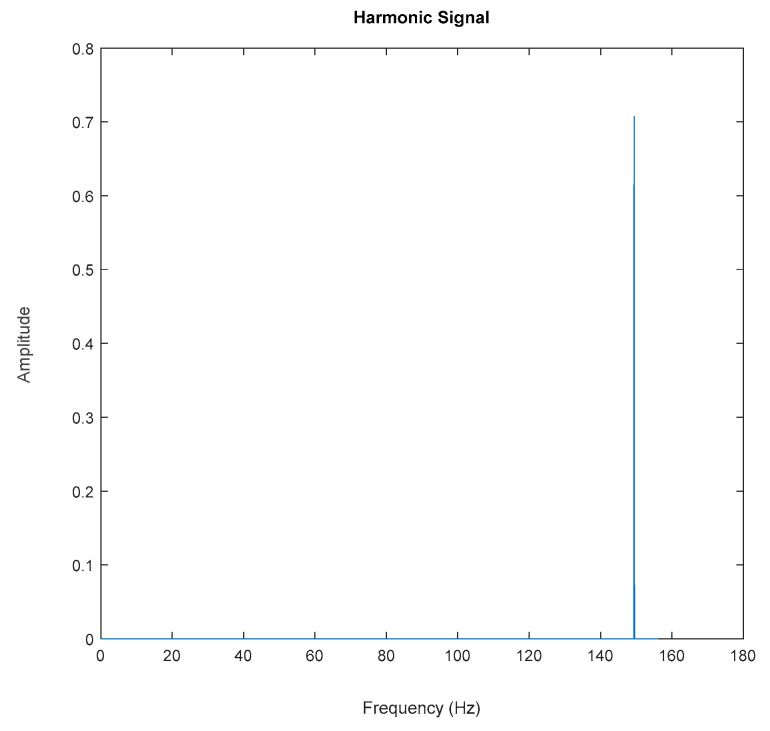
FFT of the generated harmonic signal based on estimated coefficients using iterative least-squares estimation.

Based on the image clustering result, a plot using image features extraction from Maximally Stable External Regions was displayed in Figure 28 with all the poles of the stabilization diagram that are presented in Figure 27. Using standardized image features in MATLAB, image clustering was able to capture and provide a clear appearance of all modes of interest, including structural modes and harmonic components in the stabilization diagram, as characterized in Table 15 and Figure 29. Meanwhile, the result of image shape recognition for the harmonic components is shown in Table 16. Knowing the frequency that represents noise mode or harmonic component is essential because it can be used for the next step to reconstruct a sinusoidal signal that represents harmonic frequency by using a sinusoidal model fitting, of which the results are displayed in Figure 34 and Figure 35. Then, the generated sinusoidal signals are automatically removed from the original signal to become a clean input time-series signal that is free from the harmonic component. The results after going through the harmonic removal process can be clearly seen in Figure 30, Figure 31, Figure 32 and Figure 33, where only structural poles are left, and they are free from harmonic components poles.

In order to demonstrate the novel ability of the proposed SSI-Covariance with the image-based feature extraction technique to eliminate harmonics in realistic cases, modal parameters are also analyzed. The proposed method is validated by using stochastic subspace identification-data (SSI-Data), one of the well-known time-domain methods, mainly due to how robust and powerful it is in extracting structural modal parameters. Then, the proposed algorithm is also compared with the classical SSI-Covariance algorithm. Dealing with real or complex structures faces difficulties, especially in validating data for output-only data by using floor mass and stiffness properties of the structure. Besides that, the use of the EMA method with complete input-output data to validate data for the whole structure seems to impose additional challenges as the forced vibration testing requires the use of controlled and measurable dynamic excitation, which is very heavy and expensive devices for artificially generated vibrations. This may also cause damage to a structure when subjected to severe dynamic loading. In addition, the dynamic properties of a structure at a low level of vibration may be different compared to artificially generated vibrations which are normally between 10% and 20% for the natural periods of vibration but can exceed 30% for the reinforced concrete structure due to the crack of the structural elements [31]. The best way to validate data is to conduct comparative studies with different techniques with respect to modal parameters which will produce some confidence to have a good estimation of the real system. The attempted simulations have confirmed the efficacy of these two approaches to crosscheck along with the effectiveness of the proposed algorithm on the steel frame during experimental testing. All SSI versions use the same 50 number of model orders for the estimation.

**Figure 36:**
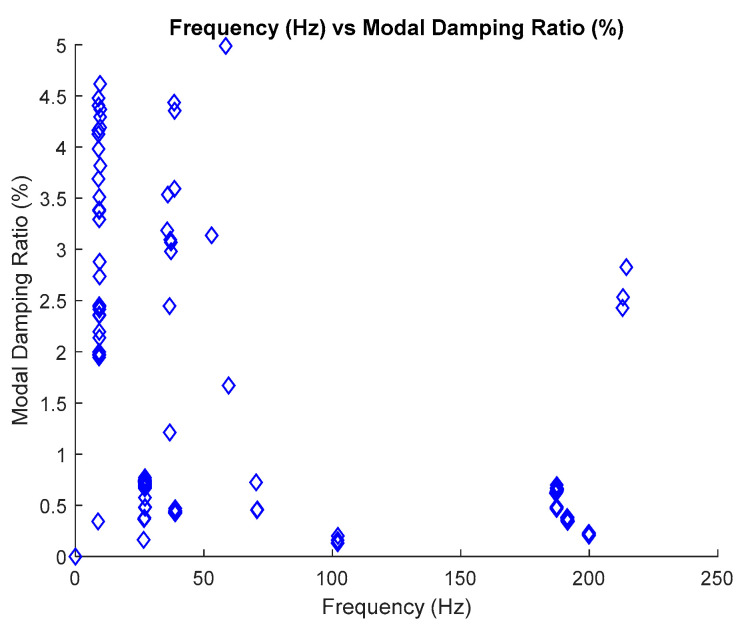
Plot frequency versus modal damping ratio.

**Figure 37:**
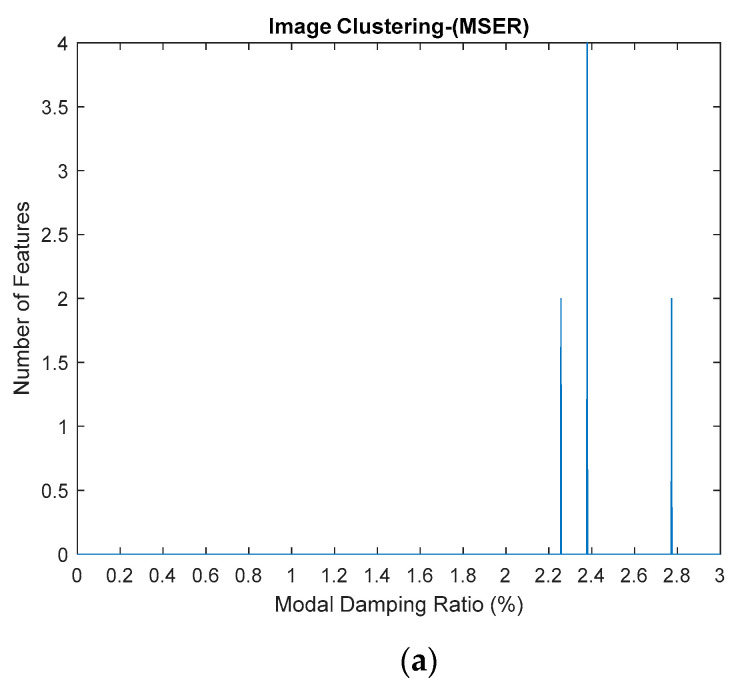

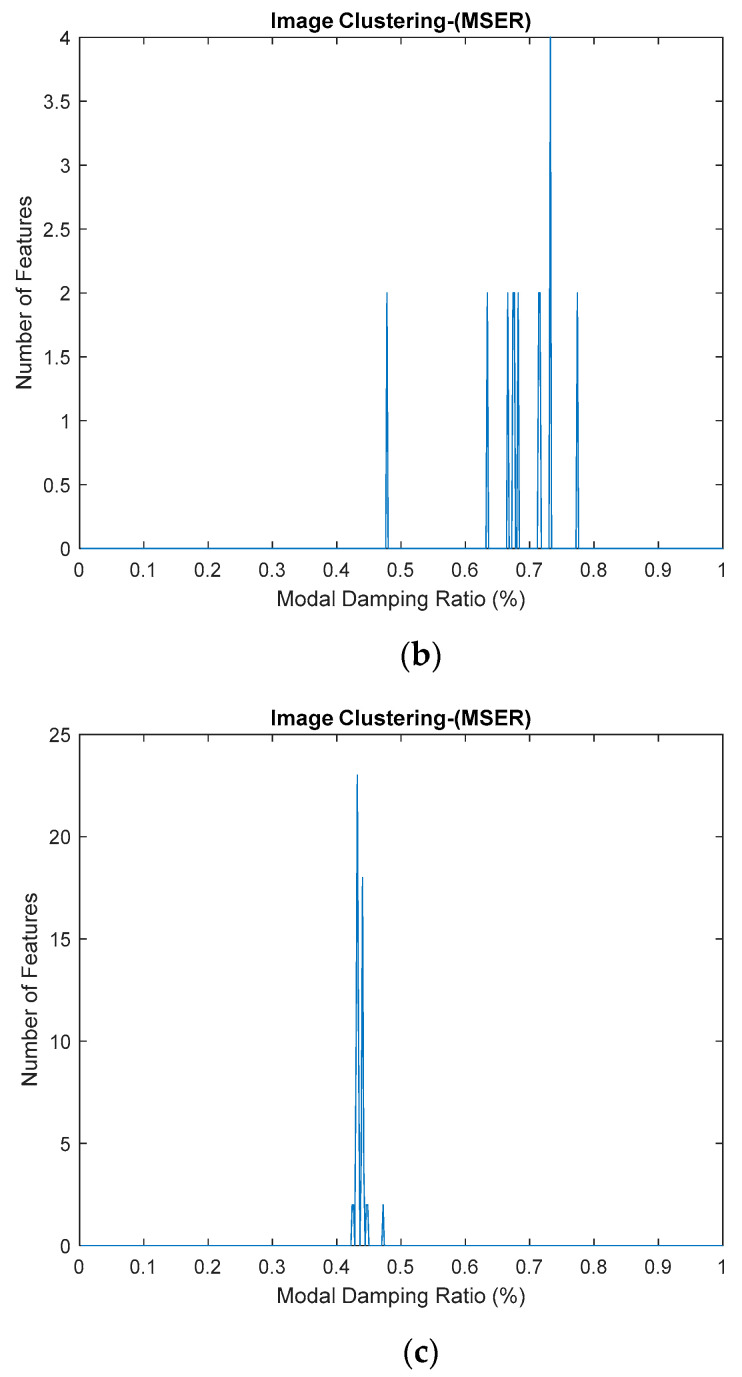
(**a**–**c**) First, second and third modes for modal damping ratios, respectively.

Results obtained from the two different approaches are also presented in Table 17, Table 18, Table 19 and Table 20 and compared with the proposed SSI-Covariance with the image-based feature extraction technique. Table 17 and Table 18 provide the results of estimated natural frequency before and after removing the harmonic components, respectively. Meanwhile, Table 19 and Table 20 indicate the results of estimated modal damping ratios for before and after removing the harmonic components, respectively. Detailed results of modal damping ratios displayed in the image clustering plot for each mode are also presented in Figure 37, which have been generated from the frequency versus modal damping ratio plot in Figure 36. By comparing the results of the estimated modal parameter using the proposed automated harmonic signal removal using SSI-Covariance with image-based feature extraction with other SSI methods, which are classical SSI-Covariance and SSI-Data, results of the proposed approach in estimating natural frequencies demonstrated very high accuracy, exhibiting an average percentage deviation less than 1% for the methods. In addition, the other modal parameter, particularly the modal damping ratio, exhibited consistent results and was within the range for before and after removing the harmonic components from the response signal among comparison methods and it seems not very affected with the corrupted response signal with the unwanted mode. For the examined cases, it has been verified that the developed algorithm works well and yields clear results that can effectively handle realistic cases that consist of corrupted harmonic components as noise mode and are capable of structural health monitoring. In the literature, the modal damping ratio is considered as a good practical parameter or indicator for structural damage detection due to its sensitivity and is sufficiently responsive to damage compared to mode shape and natural frequency.

## 4. Conclusions

In this paper, an operational modal identification method was proposed for ambient vibration testing in the presence of harmonics. This operational modal identification method, based on proposed automated harmonic signal removal using SSI-Covariance with image-based feature extraction, was first applied to a three-degrees-of-freedom system in order to validate its numerical implementation, and then investigated via an experimental steel frame. Identified modal parameters were compared to those found by a classical OMA approach based on the white noise excitation assumption, namely the SSI method, classical SSI-Covariance and SSI Data. Results of the proposed approach in estimating modal parameters demonstrated very high accuracy and exhibited consistent results for before and after removing the harmonic components from the response signal compared to other comparison methods.

The main advantage of the proposed automated harmonic signal removal using SSI-Covariance with image-based feature extraction is its independence from the nature of the excitation and its novel ability to eliminate harmonic components. Consequently, it provides an accurate prediction of modal parameters in the presence of harmonic excitations. Thus, it opens possibilities for application to more complicated structures in the presence of harmonic excitations under operational conditions. The use of an image clustering rather than a normal clustering algorithm can neglect any calibration or user-defined parameter at start-up and any supplementary adaptive approach for cluster validation criteria.

In the present day, image-based vibration measurement has brought great attention to civil engineering communities and is increasingly being used in the area of structural dynamics, particularly for modal analysis and damage identification [47,48,49,50,51]. Optically acquired data, usually from digital image correlation, as an alternative method was introduced to reduce labor-intensive tasks during dynamic testing involving the multiple numbers of accelerometers and handling the wiring and the connections [51,52]. Various image-processing techniques are being used to identify the displacements from image sequences. Some of the most commonly used techniques are: Gradient-Based Optical Flow [53,54,55], Gradient-Based Digital Image Correlation [50], in fact the Lucas-Kanade method from Reference [54] is the general form of digital image correlation (DIC) [56], Point Tracking [49] and the Phase-Based method [51,57]. Existing image-based applications are mostly used to detect movement of target objects and act as virtual sensors, but in contrast to this study, the use of image-based applications involves image-based feature extraction as a new tool for clustering of physical modes and unwanted modes in the stabilization diagram and also be used for structural modal identification. This research serves as a base for enhancing the automation of the OMA method without any user interaction.

This also verifies that the developed algorithm can be used for continuous structural health monitoring by taking advantage of ambient excitation, which is always present. This allows for tracking the evolution of modal parameters over time and can be used to detect structural integrity or problems due to structural deterioration, or the occurrence of damage to the structure. For example, when the structure gets old, the value of a modal parameter such as natural frequency will reduce over time due to loss of stiffness, while the modal damping ratio will increase over time due to rusted steel. In general, the variation of natural frequencies over time is more apparent to be adopted as a parameter for damage detection due to consistent trends but, it has a somewhat low sensitivity unless severe damage happens and is reported to be a less than 5% change in frequency associated with critical damage. Conversely, the use of a modal damping ratio as a practical parameter or indicator for structural damage detection is more ideal and suitable due to its sensitivity and is sufficiently responsive to damage compared to natural frequency and mode shape, even though it is less popular among the engineering community because of inconsistent trends [1,45]. Therefore, with the effectiveness of the proposed approach in terms of removing unwanted signal from the response signal and estimate modal identification, particularly modal damping ratio, to a sufficient degree of accuracy becomes a stepping stone to achieve reliable modal estimates and to develop effective and reliable means of modal-based damage detection for locating, quantifying structural damage and obtaining maximum useful information at a minimum cost, because it can save the total cost of repairs and extends the remaining service life of the structure through early damage detection.

## Figures and Tables

**Table 1:** Overview of methods for identifying harmonic components and structural modes.

Technique	Description
Short-Time Fourier Transform (STFT)	Short-time Fourier transform (STFT) is one of the methods of linear time-frequency domain analysis and display response in a contour plot. For structural modes, the plot will indicate thick vertical lines, while harmonic components are shown as thin vertical lines for stable conditions.
Singular Value Decomposition (SVD) [19]	When SVD curves are plotted, the peaks will indicate whether they are due to a harmonic component or a structural mode. If a narrow peak is shown in more than one singular value, this will indicate a harmonic excitation, while a peak of a structural mode only appears in one singular value due to the rank of the matrix.
Visual Mode Shapes Comparison	Operating deflection shape (ODS) will be displayed with the combination of several excited modes if the frequency of a harmonic component appears far away from a structural mode. Meanwhile, if the frequency of a harmonic component is near to structural mode, the ODS of the harmonic component will be similar to the mode shape and thus can be mistaken for being a mode shape.
Modal Assurance Criterion (MAC) [20]	The MAC value plays a crucial role to determine whether they are due to a harmonic component or a structural mode, which will depend on the modes being excited. The MAC value will produce a high correlation between a true mode shape and an ODS of the harmonic component, while low correlation is generated for closely spaced modes.
Stabilization Diagram [21,22]	A stabilization diagram can distinguish between stable, unstable and noise modes based on the specific requirements of the mode indicator and which one is respected to the set for variation between models of consecutive orders thresholds. Meanwhile, a stabilization criterion for harmonic components is identified by adjusting the valid range of damping ratios.
Probability Density Functions (PDFs) [23]	The significant difference in PDF distribution of a harmonic component and stochastic input can be used as a harmonic indicator. The response of stochastic input yields a PDF distribution of Gaussian-distributed bells, while the harmonic component will produce a deterministic sinusoidal response at their excitation frequency.
Kurtosis [16,24,25,26,27]	Kurtosis criteria have also been used to identify harmonic components and structural modes based on a significant difference in the statistical properties of PDF. The kurtosis value, γ for PDF of the sinusoidal function, is 1.5 by showing it as more “pointier”. Meanwhile, a Gaussian PDF is much wider with kurtosis value γ of 3.

**Table 2:** Specification of shape and color according to the type of poles.

Type of Poles	Type of Shape	Color
Stable	Diamond	Blue
Unstable	Circle	Red
Harmonic	Square	Green

**Table 3:** The adopted parameters for the three-story frame in the processing.

Parameters	Three-Story Frame
Length of time series, t (s)	600
Sampling frequency, $f_{s}$ (Hz)	200
Adopted frequency resolution, Δf (Hz)	0.00167

**Table 4:** Application of fixed harmonic excitation on the three-shear frame model.

Components	Harmonic Frequency (Hz)
1st	3
2nd	6
3rd	9

**Table 5:** Exact modal parameter of the three-shear frame model.

Mode	Natural Frequency (Hz)	Modal Damping Ratio (%)
1st	2.657	1.00
2nd	7.44	0.84
3rd	10.76	1.00

**Table 6:** Estimated dominant frequencies of the three-shear frame model.

Components	No. of the Feature Region	No. of Figure	Identified Frequency	Nature
1st	66	267	2.67	Structural
2nd	39	301	3.01	Harmonic
3rd	40	600	6.00	Harmonic
4th	69	742	7.42	Structural
5th	40	901	9.01	Harmonic
6th	33	1065	10.65	Structural

**Table 7:** Estimated harmonic components of the three-shear frame model.

Component	Frequency (Hz)	No. of Square Shape Recognition
1st	3.01	39
2nd	6.00	40
3rd	9.01	30

**Table 8:** Estimated coefficient values for sinusoidal model fitting of harmonic components in the three-shear frame model.

Component	Target Coefficient Value		Estimated Coefficient Value	
	*A* [10^−5^]	*B*	*A* [10^−5^]	*B*
1st	1.500	3	1.491	3.01
2nd	1.500	6	1.495	6.00
3rd	1.500	9	1.505	9.01

**Table 9:** Estimated damping ratios of the three-shear frame model.

Mode	Number of the Feature Region	No. of Figure	Identified Damping Ratios (%)
1st	27	516	1.032
2nd	16	421	0.842
3rd	5	491	0.982

**Table 10:** Comparison of estimated natural frequencies of the three-shear frame model before removing the harmonic signal.

Mode	Target Natural Frequency (Hz)	Natural Frequency (Hz)					
		Proposed SSI	Error (%)	Classical SSI	Error (%)	SSI-Data	Error (%)
1st	2.657	2.67	0.49	2.67	0.49	2.67	0.49
2nd	7.445	7.42	0.34	7.41	0.47	7.42	0.34
3rd	10.759	10.65	1.01	10.66	0.92	10.66	0.92

**Table 11:** Comparison of estimated natural frequencies of the three-shear frame model after removing the harmonic signal.

Mode	Target Natural Frequency (Hz)	Natural Frequency (Hz)					
		Proposed SSI	Error (%)	Classical SSI	Error (%)	SSI-Data	Error (%)
1st	2.657	2.67	0.49	2.66	0.11	2.66	0.11
2nd	7.445	7.42	0.34	7.41	0.47	7.42	0.34
3rd	10.759	10.65	1.01	10.66	0.92	10.65	1.01

**Table 12:** Estimated modal damping ratios of the three-shear frame model before removing the harmonic signal.

Mode	Target Modal Damping Ratio (%)	Modal Damping Ratio (%)					
		Proposed SSI	Error (%)	Classical SSI	Error (%)	SSI-Data	Error (%)
1st	1.000	1.040	4.00	1.276	27.60	1.049	4.90
2nd	0.841	0.842	0.12	0.876	4.16	0.863	2.62
3rd	1.000	0.982	1.80	0.976	2.40	0.969	3.10

**Table 13:** Comparison of estimated modal damping ratios of the three-shear frame model after removing the harmonic signal.

Mode	Target Modal Damping Ratio (%)	Modal Damping Ratio (%)					
		Proposed SSI	Error (%)	Classical SSI	Error (%)	SSI-Data	Error (%)
1st	1.000	1.032	3.20	0.995	0.50	0.983	1.70
2nd	0.841	0.842	0.12	0.879	4.52	0.861	2.38
3rd	1.000	0.982	1.80	0.973	2.70	0.968	3.2

**Table 14:** The adopted parameters for the steel frame in the processing.

Parameters	Steel Frame
Length of time series, t (s)	300
Sampling frequency, $f_{s}$ (Hz)	640
Adopted frequency resolution, Δf (Hz)	0.0033

**Table 15:** Estimated dominant frequencies of the steel frame.

Components	No. of the Feature Region	No. of Figure	Identified Frequency	Nature
1st	12	927	9.27	Structural
2nd	24	2699	26.99	Structural
3rd	71	3884	38.84	Structural
4th	36	14971	149.71	Harmonic

**Table 16:** Estimated harmonic components of the steel frame.

Component	Frequency (Hz)	Number of Square Shape Recognition
1st	149.71	36

**Table 17:** Comparison of estimated natural frequencies of the steel frame before removing the harmonic signal.

Mode	Natural Frequency (Hz)		
	Proposed SSI	Classical SSI	SSI-Data
1st	9.27	9.28	9.27
2nd	26.99	26.99	26.99
3rd	38.84	38.83	38.83

**Table 18:** Comparison of estimated natural frequencies of the steel frame after removing the harmonic signal.

Mode	Natural Frequency (Hz)		
	Proposed SSI	Classical SSI	SSI-Data
1st	9.27	9.28	9.27
2nd	27.05	26.99	26.99
3rd	38.84	38.83	38.83

**Table 19:** Comparison of estimated modal damping ratios of the steel frame before removing the harmonic signal.

Mode	Modal Damping Ratio (%)		
	Proposed SSI	Classical SSI	SSI-Data
1st	2.380	2.633	2.468
2nd	0.748	0.729	0.714
3rd	0.432	0.427	0.426

**Table 20:** Comparison of estimated modal damping ratios of the steel frame after removing the harmonic signal.

Mode	Modal Damping Ratio (%)		
	Proposed SSI	Classical SSI	SSI-Data
1st	2.378	2.633	2.468
2nd	0.732	0.729	0.714
3rd	0.432	0.427	0.426

**Table 21:** Estimated damping ratios of the steel frame.

Mode	No. of the Feature Region	No. of Figure	Identified Damping Ratios (%)
1st	4	1189	2.378
2nd	4	366	0.732
3rd	23	216	0.432

**Figure A1:**
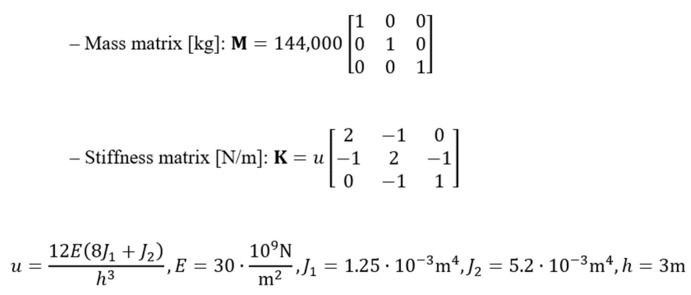
The main features of the three-story frame.

## Appendix A

Mass and stiffness matrices are set fixed for three-story frame as reported below. Damping matrices have been assumed to be diagonal in modal coordinates and represented by different modal damping ratios in the numerical tests. Dynamic system models: main features of ideal multi shear-type frames is shown in Figure A1 below.
